# Degree of hypertension and subclinical coronary atherosclerosis in asymptomatic individuals without cardiovascular disease

**DOI:** 10.1371/journal.pone.0353359

**Published:** 2026-07-10

**Authors:** Mi-Hee Jang, Sangwoo Park, Soe Hee Ann, Yong-Giun Kim, Young-Jee Jeon, Soyeoun Lim, Woon-Jung Kwon, Seong Hoon Choi, Hyun Woo Park, Gyung-Min Park

**Affiliations:** 1 Department of Cardiology, Ulsan University Hospital, University of Ulsan College of Medicine, Ulsan, Republic of Korea; 2 Department of Family Medicine, Ulsan University Hospital, University of Ulsan College of Medicine, Ulsan, Republic of Korea; 3 Department of Radiology, Ulsan University Hospital, University of Ulsan College of Medicine, Ulsan, Republic of Korea; 4 Department of Cardiology, Soonchunhyang University Bucheon Hospital, Soonchunhyang University College of Medicine, Bucheon, Republic of Korea; 5 Basic-Clinic Convergence Research Center, University of Ulsan, Ulsan, Republic of Korea; Instituto Nacional de Cardiologia Ignacio Chavez, MEXICO

## Abstract

**Objective:**

This study sought to evaluate the association between degree of hypertension and subclinical coronary atherosclerosis.

**Design and method:**

We analyzed 7,332 asymptomatic individuals (mean age 52.8 ± 7.8 years; 4,680 [63.8%] men) without cardiovascular disease who voluntarily underwent coronary computed tomography angiography (CCTA) as part of a general health examination. Hypertension classification was adapted from the American College of Cardiology/American Heart Association 2025 guidelines. The degree of coronary artery disease (CAD) was evaluated using CCTA and classified as normal coronary arteries, non-obstructive CAD (diameter stenosis <50%), and obstructive CAD (diameter stenosis ≥50%).

**Results:**

The participants were classified into 4 groups: normal (systolic blood pressure [SBP] <120 mmHg and diastolic blood pressure [DBP] <80 mmHg; n = 2,500), elevated (SBP 120–129 mmHg and DBP < 80 mmHg; n = 969), stage 1 hypertension (SBP 130–139 mmHg or DBP 80–89 mmHg; n = 2,841), and stage 2 hypertension (SBP ≥ 140 mmHg or DBP ≥ 90 mmHg; n = 1,022). After adjusting for cardiovascular risk factors, the stage 1 hypertension group was significantly associated with non-obstructive CAD (adjusted odds ratio [aOR] 1.335; 95% confidence interval [CI], 1.156–1.541). Furthermore, the stage 2 hypertension group had a significant association with both non-obstructive CAD (aOR, 1.483; 95% CI, 1.234–1.784) and obstructive CAD (aOR, 1.696; 95% CI, 1.194–2.409).

**Conclusions:**

In this large cross-sectional study, the degree of hypertension was associated with an increased risk and severity of subclinical coronary atherosclerosis. These findings highlight the potential importance of early recognition of blood pressure elevation and cardiovascular risk stratification in asymptomatic individuals.

## 1. Introduction

Subclinical atherosclerosis is characterized by atherosclerotic changes in the arterial wall in the absence of clinical manifestations of cardiovascular disease [1]. It is prevalent among individuals with traditional cardiovascular risk factors such as hypertension, dyslipidemia, diabetes and smoking, and is recognized as a strong predictor of future cardiovascular events [2]. As subclinical atherosclerosis precedes clinically manifest cardiovascular disease, identifying its determinants is critical for improving risk stratification and guiding early intervention [3–6]. Coronary computed tomography angiography (CCTA) is the standard of care for diagnosing obstructive coronary artery disease (CAD) in symptomatic patients and is increasingly acknowledged as a reliable non-invasive modality for detecting subclinical coronary atherosclerosis and predicting long-term outcomes [7–10].

Blood pressure (BP) is a well-established and modifiable determinant of cardiovascular risk, consistently shown to have a linear association with cardiovascular events [11,12]. Recent trials have demonstrated that intensive blood pressure control reduces the risk of atherosclerotic cardiovascular disease (ASCVD), leading current guidelines to recommend lower BP targets [13–16]. In particular, pharmacologic therapy is recommended for individuals with systolic BP of 130–139 mmHg when additional risk factors are present, whereas lifestyle modification remains the primary approach for those at lower risk [14,16]. However, large-scale imaging evidence evaluating subclinical coronary atherosclerosis across contemporary BP categories in asymptomatic screening populations remain limited. Therefore, in this study, we aimed to evaluate the association between the degree of hypertension and the presence and severity of subclinical coronary atherosclerosis in a large cohort of asymptomatic individuals who underwent CCTA during a health check-up.

## 2. Methods

### 2.1. Study population

**Fig 1:**
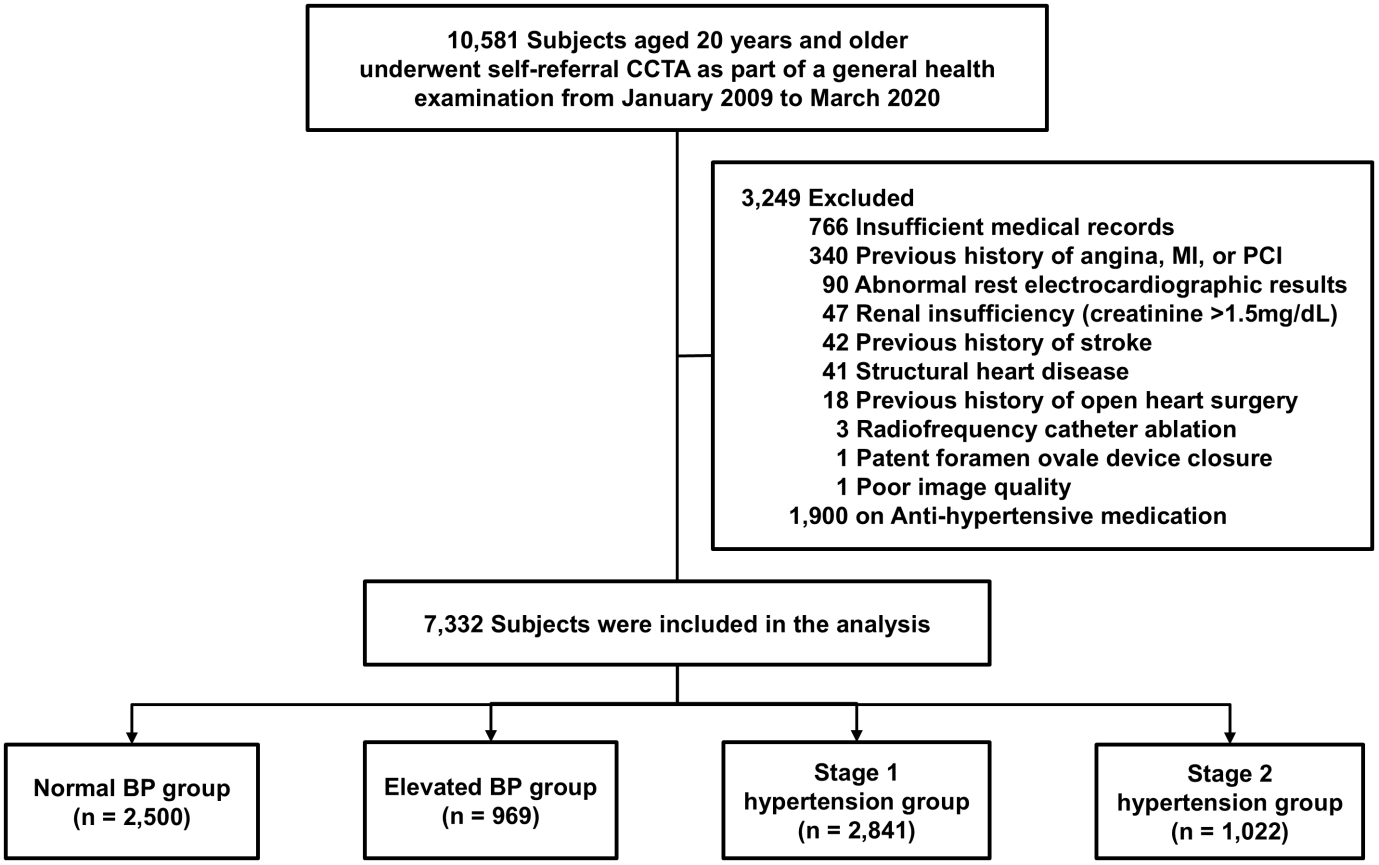
Study flowchart. The flowchart shows the subject selection process. The numbers of subjects excluded and enrolled in the current study are shown. BP = blood pressure; CCTA = coronary computed tomographic angiography; MI = myocardial infarction; PCI = percutaneous coronary intervention.

South Korea has a National Health Insurance system. The National Health Insurance in South Korea has actively operated the business of promoting health checkups and health levels in an effort to detect diseases early and enhance public health. These general health screenings have been performed on the employee insured with no age limit (annually) and the self-employed insured, such as householders or dependents aged >40 years (biannually). These general checkups are free and covered by the National Health Insurance. In addition, if individuals undergoing general medical checkups paid additional costs, they could take additional tests, such as CCTA. In this study, a total of 10,581 individuals aged ≥20 years who voluntarily underwent CCTA as part of a general health examination at the Health Promotion Center of Ulsan University Hospital (Ulsan, South Korea) between January 2009 and March 2020 were retrospectively included in the study. Inclusion criteria were (1) age ≥ 20 years and (2) asymptomatic status at the time of CCTA. Exclusion criteria were (1) insufficient medical records (n = 766), (2) history of angina or myocardial infarction and/or percutaneous coronary intervention (n = 340), (3) abnormal 12-lead electrocardiography (ECG) results (n = 90), (4) renal insufficiency (serum creatinine > 1.5 mg/dL) (n = 47), (5) previous history of stroke (n = 42), (6) structural heart disease (n = 41), (7) history of open heart surgery (n = 18), (8) history of radiofrequency catheter ablation (n = 3), (9) history of patent foramen ovale device closure (n = 1), (10) poor image quality (n = 1), and (11) on anti-hypertensive medication (n = 1,900). After applying these criteria, 7,332 participants were included in the final analysis. The participant enrollment process is shown in **Fig 1**. This study was approved by the Institutional Review Board of Ulsan University Hospital (Ulsan, Korea), and the requirement for informed consent was waived due to its retrospective and cross-sectional design (IRB No. UUH 2020-12-033). All analyses were conducted using de-identified clinical data obtained from medical records. The dataset was first accessed for research purposes on September 30, 2025, and the final data analysis was completed on October 31, 2025.

### 2.2. Clinical and laboratory measurements

We collected clinical and laboratory data from electronic medical records and the clinical data warehouse platform at Ulsan University Hospital. During routine medical examinations, height, body weight, and waist circumference were measured according to standard procedures. After overnight fasting, blood samples were collected to assess fasting blood glucose, hemoglobin A1c, total cholesterol, low-density lipoprotein cholesterol (LDL-C), high-density lipoprotein cholesterol (HDL-C), triglycerides, creatinine, and uric acid levels. All participants underwent standard 12-lead electrocardiography. Transthoracic echocardiography was performed to determine the left ventricular ejection fraction. Diabetes mellitus (DM) was defined as a fasting plasma glucose level of ≥ 126 mg/dL, hemoglobin A1c of ≥ 6.5%, or a self-reported history of diabetes and/or treatment with dietary changes or anti-diabetic medication. A body mass index of ≥ 25 kg/m^2^ was used as the World Health Organization’s Asian-specific criterion for obesity. Total cholesterol levels of ≥ 240 mg/dL, self-reported history of dyslipidemia, and/or use of antihyperlipidemic medication were considered indicators of dyslipidemia [17]. A family history of CAD was noted if a first-degree relative of any age had CAD [18]. The 10-year ASCVD risk score was calculated using pooled cohort equations [19].

### 2.3. Blood Pressure Measurement and classification

BP was measured by trained examiners after a rest period of at least 5 min, using an automatic manometer and a properly sized cuff. Systolic and diastolic BPs were recorded three times, and the average of the second and third measurements was used for analysis [20]. For our study, BP classification was based on the American College of Cardiology/American Heart Association 2025 guidelines: normal (systolic blood pressure [SBP] < 120 mmHg and diastolic blood pressure [DBP] < 80 mmHg), elevated (SBP 120–129 mmHg and DBP < 80 mmHg), stage 1 hypertension (SBP 130–139 mmHg or DBP 80–89 mmHg), and stage 2 hypertension (SBP ≥ 140 mmHg or DBP ≥ 90 mmHg) [16].

### 2.4. Coronary computed tomography angiography image acquisition and analysis

CCTA was conducted using either a single-source 256-slice CT scanner (Brilliance iCT; Philips Healthcare, Best, Netherlands) or a dual-source system (Somatom Definition Flash; Siemens, Erlangen, Germany). A standardized imaging protocol was applied, as previously described [17]. Image interpretation and coronary calcium scoring were independently performed by three cardiovascular imaging experts, two radiologists and one cardiologist (W.J.K., S.H.C., and G.M.P., each with over 10 years of experience), who were blinded to the clinical information. CCTA scans were analyzed using dedicated workstations (Syngo.via, Siemens or Aquarius iNtuition, Terarecon). Final interpretations were made by consensus. Coronary artery calcium scores (CACS) were evaluated and categorized based on the Agatston method into the following groups: 0, 1–10, 11–100, 101–400, and > 400 [21]. The degree of luminal narrowing was assessed by semi-automatically measuring the contrast-enhanced lumen at the site of maximal stenosis and comparing it with the average diameter of adjacent proximal and distal reference segments. Stenosis of ≥50% was defined as obstructive CAD, whereas stenosis of <50% was classified as non-obstructive CAD [22].

### 2.5. Statistical analysis

Categorical variables are presented as frequencies and percentages. Continuous variables are presented as mean ± standard deviation or median (interquartile range), as appropriate. Pearson’s chi-square test or Fisher’s exact test was used to compare categorical variables, whereas continuous variables were evaluated using one-way analysis of variance or Kruskal-Wallis test, as appropriate for between-group comparisons. Univariable and multivariable logistic regression analyses were conducted to assess the association between the degree of hypertension and the risk of subclinical coronary atherosclerosis identified on CCTA. The covariates included in the multivariable model were selected based on clinical relevance and statistical significance [19,23,24], comprising age, sex, obesity, diabetes mellitus, dyslipidemia, current smoking, and family history of CAD. Both unadjusted and adjusted odds ratios (ORs) with 95% confidence intervals (CIs) were calculated. All p-values were two-sided, and a p-value <0.05 was considered statistically significant. Statistical analyses were performed using the SPSS software (version 27.0; IBM Corp., Armonk, NY, USA).

## 3. Results

### 3.1. Baseline characteristics

The average age of the population was 52.8 ± 7.8 years, and 4,680 (63.8%) participants were men. The baseline characteristics of the study cohort, stratified by the degree of hypertension, are detailed in **Table 1**. According to the 2025 American College of Cardiology/American Heart Association guidelines, participants were classified into the following categories: normal, 2,500 (34.1%); elevated, 969 (13.2%); stage 1 hypertension, 2,841 (38.7%); and stage 2 hypertension, 1,022 (13.9%). As the degree of hypertension increased, there was a tendency for higher body mass index, waist circumference, DM, obesity, fasting blood glucose, hemoglobin A1c, total cholesterol, triglycerides, uric acid, and ASCVD risk score values.

**Table 1:** Baseline characteristics of the study population according to the degree of hypertension.

Characteristics	The category of blood pressure					
	Overall (n = 7,332)	Normal (n = 2,500)	Elevated (n = 969)	Stage 1 Hypertension (n = 2,841)	Stage 2 Hypertension (n = 1,022)	p-value
Age, years	52.8 ± 7.8	51.9 ± 7.5	53.8 ± 7.7	53.1 ± 7.8	53.7 ± 8.4	<0.001
Men, no. (%)	4,680 (63.8)	1,276 (51.0)	608 (62.7)	2,092 (73.6)	704 (68.9)	<0.001
Systolic blood pressure, mmHg	123.8 ± 13.8	109.7 ± 7.2	123.9 ± 2.9	128.6 ± 7.0	144.8 ± 10.6	<0.001
Diastolic blood pressure, mmHg	78.0 ± 9.5	69.7 ± 6.1	73.7 ± 4.6	82.1 ± 4.5	91.4 ± 8.2	<0.001
Body mass index, kg/m^2^	23.9 ± 2.9	22.9 ± 2.6	24.0 ± 2.6	24.4 ± 2.7	25.2 ± 3.2	<0.001
Waist circumference, cm	84.9 ± 7.6	82.1 ± 7.1	85.2 ± 7.1	86.1 ± 7.2	87.9 ± 8.2	<0.001
Diabetes mellitus, no. (%)	750 (10.2)	189 (7.6)	100 (10.3)	324 (11.4)	137 (13.4)	<0.001
dyslipidemia, no. (%)	1,285 (17.5)	374 (15.0)	201 (20.7)	522 (18.4)	188 (18.4)	0.001
Current smoker, no. (%)	1,647 (22.9)	531 (21.6)	207 (21.8)	696 (24.9)	213 (21.2)	0.010
Obesity, no. (%)	2,392 (32.8)	497 (19.9)	309 (32.2)	1,090 (38.5)	496 (48.9)	<0.001
Family history of coronary artery disease^*^	660 (9.0)	212 (8.5)	93 (9.6)	258 (9.1)	97 (9.5)	0.673
Fasting blood glucose, mg/dL	91 (84-99)	89 (82-96)	91 (84-99)	92 (85-101)	94 (85.5-104)	<0.001
Hemoglobin A1c, %	5.5 (5.3-5.8)	5.4 (5.2-5.7)	5.5 (5.3-5.8)	5.5 (5.3-5.8)	5.6 (5.3-5.9)	<0.001
Total cholesterol, mg/dL	193.8 ± 36.5	192.2 ± 34.7	193.6 ± 39.3	194.2 ± 36.7	197.0 ± 37.4	0.012
LDL cholesterol, mg/dL	129.8 ± 34.1	126.6 ± 32.4	131.9 ± 36.5	130.5 ± 34.0	133.7 ± 34.9	<0.001
HDL cholesterol, mg/dL	53.4 ± 14.9	55.4 ± 15.1	52.8 ± 14.5	52.4 ± 15.0	51.8 ± 14.4	<0.001
Triglyceride, mg/dL	94 (66-138)	85 (60-121)	94 (66-134)	102 (71-149)	105 (71-152)	<0.001
Creatinine, mg/dL	0.84 ± 0.18	0.82 ± 0.18	0.83 ± 0.18	0.87 ± 0.17	0.85 ± 0.19	<0.001
Uric acid, mg/dL	5.33 ± 1.34	5.05 ± 1.28	5.27 ± 1.29	5.49 ± 1.34	5.63 ± 1.40	<0.001
Ejection fraction, %	64.3 ± 4.5	64.5 ± 4.3	64.0 ± 5.6	64.3 ± 4.2	64.0 ± 4.9	0.048
ASCVD risk score, mean ± SD	5.7 ± 5.7	3.7 ± 4.1	5.8 ± 5.5	6.5 ± 5.8	8.1 ± 7.5	<0.001
ASCVD risk score, median (IQR)	3.9 (1.6-7.7)	2.2(0.9-5.0)	4.1(1.9-7.9)	4.9(2.3-8.7)	5.9(3.0-11.0)	<0.001

Values are shown as mean ± standard deviation, median (interquartile range), or number (%), as appropriate.

* Coronary artery disease in a ﬁrst-degree relative of any age.

ASCVD = atherosclerotic cardiovascular disease; HDL = high density lipoprotein; LDL = low density lipoprotein

### 3.2. Coronary computed tomography angiography findings

**Table 2** describes the characteristics of the CCTA findings according to the degree of hypertension. As the stage of hypertension increased, the mean CACS also increased (normal versus stage 2 hypertension; 15.8 ± 90.1 versus 50.2 ± 190.5, p < 0.001). A statistically significant increase in the proportion of participants with higher CACS categories was observed with higher degrees of hypertension. Additionally, the degree of hypertension was associated with a trend toward a higher prevalence of both non-obstructive and obstructive CAD, with the highest prevalence observed in patients with stage 2 hypertension (p < 0.05 for all).

**Table 2:** Comparison of coronary computed tomography angiographic findings according to the degree of hypertension.

Variables	The category of blood pressure					
	Overall (n = 7,332)	Normal (n = 2,500)	Elevated (n = 969)	Stage 1 Hypertension (n = 2,841)	Stage 2 Hypertension (n = 1,022)	p-value
Coronary artery calcium score, mean ± SD	27.3 ± 122.2	15.8 ± 90.1	23.5 ± 100.9	30.5 ± 120.6	50.2 ± 190.5	<0.001
Coronary artery calcium score, median (IQR)	0 (0-1.6)	0 (0)	0 (0-1.0)	0 (0-5.5)	0 (0-12.0)	<0.001
Coronary artery calcium score, no. (%)						<0.001
0	5,356 (73.1)	2,025 (81.1)	722 (74.5)	1,951 (68.7)	658 (64.4)	
1-10	587 (8.0)	146 (5.8)	72 (7.4)	275 (9.7)	94 (9.2)	
11-100	892 (12.2)	230 (9.2)	118 (12.2)	386 (13.6)	158 (15.5)	
101-400	388 (5.3)	77 (3.1)	46 (4.7)	183 (6.4)	82 (8.0)	
>400	105 (1.4)	20 (0.8)	11 (1.1)	44 (1.5)	30 (2.9)	
Severity of subclinical coronary atherosclerosis						<0.001
Normal	5,235 (71.4)	1,984 (79.4)	708 (73.1)	1,900 (66.9)	643 (62.9)	
Non-obstructive CAD	1,743 (23.8)	437 (17.5)	211 (21.8)	787 (27.7)	308 (30.1)	
Obstructive CAD	354 (4.8)	79 (3.2)	50 (5.2)	154 (5.4)	71 (6.9)	

Values are shown as mean ± standard deviation, median (interquartile range), or number (%), as appropriate.

CAD = coronary artery disease

### 3.3. Association between degree of hypertension and subclinical coronary atherosclerosis

The relationship between the degree of hypertension and subclinical coronary atherosclerosis is shown in **Table 3**. In the univariable logistic regression analysis, elevated, stage 1 and stage 2 hypertension were significantly associated with an increased risk of non-obstructive and obstructive CAD. After adjusting for cardiovascular risk factors, including age, sex, obesity, diabetes mellitus, dyslipidemia, current smoking, and family history of coronary artery disease, non-obstructive CAD was significantly associated with stage 1 hypertension (adjusted OR 1.335, 95% CI 1.156–1.541; p < 0.001) and stage 2 hypertension (1.483, 95% CI 1.234–1.784; p < 0.001). Furthermore, the adjusted ORs for obstructive CAD were not significantly associated with stage 1 hypertension (1.261, 95% CI 0.940–1.692, p = 0.122) but were significantly higher in stage 2 hypertension (1.696, 95% CI 1.194–2.409; p = 0.003). Additionally, when coronary artery calcium burden was evaluated, stage 1 hypertension was associated with CACS ≥ 1 and > 100 but not CACS > 400. On the other hand, stage 2 hypertension remained a significant association with CACS ≥ 1, > 100, and > 400 (**Table 4**).

**Table 3:** Univariable and multivariable analyses for the association between degree of hypertension and subclinical coronary atherosclerosis.

Variables	Non-obstructive CAD				Obstructive CAD			
	Univariable analysis		Multivariable analysis		Univariable analysis		Multivariable analysis	
	Odds ratio (95% CI)	p-value	Odds ratio (95% CI)	p-value	Odds ratio (95% CI)	p-value	Odds ratio (95% CI)	p-value
Age	1.073 (1.065–1.081)	<0.001	1.085 (1.076–1.094)	<0.001	1.105 (1.089–1.120)	<0.001	1.115 (1.098–1.132)	<0.001
Male sex	2.886 (2.535–3.285)	<0.001	2.880 (2.488–3.334)	<0.001	3.449 (2.560–4.648)	<0.001	3.713 (2.691–5.123)	<0.001
Obesity	1.454 (1.300–1.626)	<0.001	1.295 (1.146–1.465)	<0.001	1.143 (0.913–1.430)	0.243		
Diabetes mellitus	2.139 (1.825–2.506)	<0.001	1.542 (1.299–1.830)	<0.001	3.050 (2.362–3.939)	<0.001	2.233 (1.702–2.930)	<0.001
Dyslipidemia	1.389 (1.214–1.589)	<0.001	1.451 (1.256–1.677)	<0.001	1.327 (1.023–1.721)	0.033	1.408 (1.070–1.852)	0.014
Current smoking	1.516 (1.340–1.715)	<0.001	1.297 (1.129–1.490)	<0.001	1.468 (1.159–1.860)	0.001	1.312 (1.014–1.697)	0.039
Family history of CAD*	1.019 (0.845–1.228)	0.847			1.190 (0.838–1.689)	0.331	1.559 (1.078–2.254)	0.018
Category of blood pressure		<0.001		<0.001		<0.001		0.033
Normal (reference)	1	–	1	–	1	–	1	–
Elevated	1.314 (1.093–1.580)	0.004	0.978 (0.802–1.193)	0.827	1.667 (1.161–2.395)	0.006	1.305 (0.894–1.906)	0.168
Stage 1	1.809 (1.585–2.064)	<0.001	1.335 (1.156–1.541)	<0.001	1.756 (1.332–2.316)	<0.001	1.261 (0.940–1.692)	0.122
Stage 2	2.036 (1.720–2.411)	<0.001	1.483 (1.234–1.784)	<0.001	2.288 (1.646–3.180)	<0.001	1.696 (1.194–2.409)	0.003

* Coronary artery disease in a first-degree relative of any age.

Covariates in the multivariable model include age, sex, obesity (body mass index ≥25 kg/m^2^), diabetes mellitus, dyslipidemia, current smoking, and family history of coronary artery disease

CAD = coronary artery disease; CI = conﬁdence interval.

**Table 4:** Univariable and multivariable analyses for the association between degree of hypertension and coronary artery calcium score.

Variables	Coronary artery calcium score ≥1			
	Univariable analysis		Multivariable analysis	
	Odds ratio (95% CI)	p-value	Odds ratio (95% CI)	p-value
Category of blood pressure		<0.001		<0.001
Normal (reference)	1	–	1	–
Elevated	1.466 (1.230-1.748)	<0.001	1.073 (0.882-1.305)	0.480
Stage 1	1.949 (1.715-2.214)	<0.001	1.399 (1.212-1.614)	<0.001
Stage 2	2.371 (2.015-2.789)	<0.001	1.746 (1.454-2.097)	<0.001
	Coronary artery calcium score >100			
Variables	Univariable analysis		Multivariable analysis	
	Odds ratio (95% CI)	p-value	Odds ratio (95% CI)	p-value
Category of blood pressure		<0.001		<0.001
Normal (reference)	1	–	1	–
Elevated	1.532 (1.096-2.142)	0.013	1.099 (0.767-1.576)	0.608
Stage 1	2.128 (1.669-2.715)	<0.001	1.511 (1.163-1.963)	0.002
Stage 2	3.017 (2.276-3.998)	<0.001	2.231 (1.640-3.034)	<0.001
	Coronary artery calcium score >400			
Variables	Univariable analysis		Multivariable analysis	
	Odds ratio (95% CI)	p-value	Odds ratio (95% CI)	p-value
Category of blood pressure		<0.001		<0.001
Normal (reference)	1	–	1	–
Elevated	1.424 (0.680-2.983)	0.349	0.971 (0.449-2.098)	0.940
Stage 1	1.951 (1.147-3.318)	0.014	1.261 (0.717-2.217)	0.421
Stage 2	3.750 (2.120-6.635)	<0.001	2.449 (1.321-4.541)	0.004

Covariates in the multivariable model include age, sex, obesity (body mass index ≥25 kg/m^2^), diabetes mellitus, dyslipidemia, current smoking, and family history of coronary artery disease

CI = conﬁdence interval.

## 4. Discussion

In this large cohort of asymptomatic individuals without a prior history of cardiovascular disease, hypertension severity was significantly associated with the prevalence of subclinical coronary atherosclerosis. Both stage 1 and stage 2 hypertension were independently associated with non-obstructive CAD, while stage 2 hypertension was additionally associated with obstructive CAD. These findings are consistent with prior epidemiological studies demonstrating a graded association between blood pressure and coronary atherosclerosis and extend this evidence through imaging-based assessment in an asymptomatic screening population [11,25]. In contrast, the association observed in the elevated BP category was attenuated after multivariable adjustment, suggesting that the independent effect of BP within this range may be limited after accounting for other cardiovascular risk factors. However, taken together, these findings highlight the potential importance of early recognition of blood pressure elevation in asymptomatic populations.

In addition to stenosis-based CAD severity, we observed a graded increase in coronary artery calcium burden across hypertension stages, including higher odds of CACS ≥ 1, > 100, and > 400 in multivariable analyses. Coronary artery calcium is a well-established marker of subclinical atherosclerosis and an important predictor of future cardiovascular risk [26]. Previous studies in asymptomatic populations have similarly reported higher coronary calcium burden with increasing blood pressure severity, although differences exist in BP classification systems and analytic approaches [27,28]. Specifically, earlier studies such as those by Im et al. applied the Seventh Report of the Joint National Committee (JNC 7) classification, whereas our study used contemporary ACC/AHA guidelines. Moreover, while prior studies mainly evaluated the presence of coronary artery calcium, we assessed coronary artery calcium burden in greater detail and further evaluated luminal stenosis severity using CCTA. Despite these differences, the overall trend is consistent, supporting the concept that greater blood pressure severity is associated with a coronary calcium burden reflecting total atherosclerotic burden.

Contemporary randomized controlled trials, including SPRINT (Systolic Blood Pressure Intervention Trial) and STEP (Strategy of Blood Pressure Intervention in the Elderly Hypertensive Patients), have demonstrated that intensive BP control significantly reduces the risk of major cardiovascular events [13,15,29,30]. Reflecting this evidence, recent guidelines now recommend lower treatment targets, with a goal SBP of 120–129 mmHg in most adults when tolerated, and further advise pharmacologic therapy in patients with stage 1 hypertension who are considered at high risk [14,16,31]. In this context, our finding that stage 1 hypertension was associated with a higher prevalence of non-obstructive CAD provides important clinical insights. Non-obstructive CAD has long been regarded as less clinically important than obstructive CAD, and clear treatment guidelines are lacking. However, accumulating evidence shows that these patients form a large at-risk group because of the high prevalence and substantial burden of cardiovascular events [32]. Furthermore, long-term follow-up studies have shown that patients with non-obstructive CAD detected by CCTA are associated with an approximately two- to three-fold higher risk of all-cause mortality, nonfatal myocardial infarction, and subsequent revascularization [6,33–36]. Our findings suggest that early hypertension may already be associated with subclinical coronary atherosclerosis and may have implications for personalized risk assessment and prevention strategies in these individuals.

In addition, in this study, even among individuals with a relatively low ASCVD risk score (mean ASCVD risk score 5.7% and 6.5% in the overall population and subjects with stage 1 hypertension, respectively), a substantial burden of subclinical CAD was observed. Current guidelines recommend pharmacologic therapy for high-risk patients with stage 1 hypertension, while lifestyle modification is advised for those at lower-risk [14,16]. This suggests that subclinical atherosclerosis may already be present even in patients traditionally classified as lower risk.

Importantly, stage 2 hypertension was independently associated with both non-obstructive and obstructive CAD, suggesting that more advanced blood pressure elevation might be associated with not only to early atherosclerotic changes but also clinically significant coronary disease. Obstructive CAD is well established as a key determinant of prognosis [10,37,38]. In a study by Cheruvu et al. from the CONFIRM (COronary CT Angiography EvaluatioN For Clinical Outcomes: An InteRnational Multicenter Registry), involving very low-risk patients followed for an average of 5 years, the rate of major adverse cardiovascular events, including all-cause death, non-fatal MI, unstable angina, or late coronary revascularization, increased stepwise from 5.6% in those without CAD to 13.3% in those with nonobstructive CAD, and further to 36.3% in those with obstructive CAD (p < 0.001) [39]. Furthermore, in a large 10-year follow-up study, CCTA assessed CAD severity (normal, nonobstructive, or obstructive) and provided robust prognostic power for predicting cardiac death and nonfatal MI, clearly delineating diverging long-term risk trajectories and substantially improving risk reclassification [37]. Taken together, our findings suggest that increasing hypertension severity is associated with a greater burden of coronary atherosclerosis, highlighting the importance of early recognition of elevated blood pressure.

This study has several limitations. First, this was a single-center study conducted on Korean individuals, which limits the generalizability of the findings to other ethnic groups. Second, because the study population consisted of individuals who voluntarily underwent health screening with CCTA, a degree of selection bias may be inevitable, and the results may not be fully generalizable to the general population. Third, given the cross-sectional design, causal relationships cannot be inferred, and residual confounding cannot be fully excluded, despite multivariable adjustment. Fourth, the presence of coronary artery calcification may have led to an overestimation of the atherosclerotic burden, particularly when evaluating the severity of coronary artery stenosis. Fifth, our study did not include detailed high-risk plaque characteristics, such as low-attenuation plaque or positive remodeling. Due to the retrospective design and limited availability of raw CCTA imaging data, comprehensive plaque phenotyping was not feasible. Future prospective studies with detailed quantitative and qualitative plaque analysis are warranted. Sixth, blood pressure was assessed during a single health screening visit. Although systolic and diastolic blood pressures were measured three times and the average of the second and third readings was used to improve reliability, single-visit measurements may not fully reflect usual blood pressure levels. Furthermore, out-of-office measurements such as home blood pressure or ambulatory blood pressure monitoring were not available. Therefore, the possibility of white coat hypertension or masked hypertension cannot be excluded. In addition, the recruitment period of this study spanned more than a decade (2009–2020), during which changes in preventive cardiology practices and CCTA technology may have influenced the detection and classification of coronary atherosclerosis.

## 5. Conclusion

In conclusion, the degree of hypertension was significantly associated with the presence and severity of subclinical coronary atherosclerosis in asymptomatic individuals. These findings suggest that even early hypertension may be associated with subclinical coronary atherosclerosis, highlighting the potential importance of early recognition of blood pressure elevation and appropriate cardiovascular risk stratification.

## Abbreviations

ASCVD: Atherosclerotic cardiovascular disease
BP: Blood pressure
CACS: Coronary artery calcium score
CAD: Coronary artery disease
CCTA: Coronary computed tomography angiography
CI: Confidence interval
DM: Diabetes mellitus
HDL-C: High-density lipoprotein cholesterol
LDL-C: Low-density lipoprotein cholesterol
OR: Odds ratio
