# Supplementary material for: Degree of hypertension and subclinical coronary atherosclerosis in asymptomatic individuals without cardiovascular disease
Source: PLoS One. 2026 Jul 10;21(7):e0353359. doi: 10.1371/journal.pone.0353359 (PMC13354002; doi:10.1371/journal.pone.0353359)
Supplement: Attachment — Submitted filename: Response to Reviewers.docx [file pone.0353359.s002.docx]

**Journal requirements:**

**When submitting your revision, we need you to address these additional requirements.**

**1. Please ensure that your manuscript meets PLOS ONE's style requirements, including those for file naming. The PLOS ONE style templates can be found at**

[**https://journals.plos.org/plosone/s/file?id=wjVg/PLOSOne_formatting_sample_main_body.pdf**](https://journals.plos.org/plosone/s/file?id=wjVg/PLOSOne_formatting_sample_main_body.pdf) **and**

[**https://journals.plos.org/plosone/s/file?id=ba62/PLOSOne_formatting_sample_title_authors_affiliations.pdf**](https://journals.plos.org/plosone/s/file?id=ba62/PLOSOne_formatting_sample_title_authors_affiliations.pdf)

**Author’s response:**

Thank you for the Editor’s comment. We have carefully reviewed the PLOS ONE formatting guidelines and ensured that the revised manuscript complies with the journal’s style requirements.

**2. Thank you for stating the following in the Funding Section of your manuscript:**

**“This research was supported by grants of the Ulsan University Hospital Research Grant (UUH-2024-11) and the Korea Health Technology R&D Project through the Korea Health Industry Development Institute (KHIDI), funded by the Ministry of Health & Welfare, Republic of Korea (HI23C0896). Additional support was provided by the Medical Data-Driven Hospital Support Project through the Korea Health Information Service (KHIS), funded by the Ministry of Health & Welfare, Republic of Korea. The funders had no role in the study design, data collection and analysis, decision to publish, or preparation of the manuscript.”**

**We note that you have provided funding information that is currently declared in your Funding Statement. However, funding information should not appear in the Acknowledgments section or other areas of your manuscript. We will only publish funding information present in the Funding Statement section of the online submission form.**

**Please remove any funding-related text from the manuscript and let us know how you would like to update your Funding Statement. Currently, your Funding Statement reads as follows:**

**“This research was supported by grants of the Ulsan University Hospital Research Grant (UUH-2024-11) and the Korea Health Technology R&D Project through the Korea Health Industry Development Institute (KHIDI), funded by the Ministry of Health & Welfare, Republic of Korea (HI23C0896). Additional support was provided by the Medical Data-Driven Hospital Support Project through the Korea Health Information Service (KHIS), funded by the Ministry of Health & Welfare, Republic of Korea. The funders had no role in the study design, data collection and analysis, decision to publish, or preparation of the manuscript.”**

**Please include your amended statements within your cover letter; we will change the online submission form on your behalf.**

**Author’s response:**

Thank you for the Editor’s comment. In response, we have removed funding-related text from the manuscript and provided the amended Funding Statement in the cover letter, as requested.

**3. We note that you have indicated that there are restrictions to data sharing for this study. For studies involving human research participant data or other sensitive data, we encourage authors to share de-identified or anonymized data. However, when data cannot be publicly shared for ethical reasons, we allow authors to make their data sets available upon request. For information on unacceptable data access restrictions, please see** [**http://journals.plos.org/plosone/s/data-availability#loc-unacceptable-data-access-restrictions.**](http://journals.plos.org/plosone/s/data-availability#loc-unacceptable-data-access-restrictions.)

Before we proceed with your manuscript, please address the following prompts:

**a) If there are ethical or legal restrictions on sharing a de-identified data set, please explain them in detail (e.g., data contain potentially identifying or sensitive patient information, data are owned by a third-party organization, etc.) and who has imposed them (e.g., a Research Ethics Committee or Institutional Review Board, etc.). Please also provide contact information for a data access committee, ethics committee, or other institutional body to which data requests may be sent.**

b) If there are no restrictions, please upload the minimal anonymized data set necessary to replicate your study findings to a stable, public repository and provide us with the relevant URLs, DOIs, or accession numbers. Please see <http://www.bmj.com/content/340/bmj.c181.long> for guidelines on how to de-identify and prepare clinical data for publication. For a list of recommended repositories, please see <https://journals.plos.org/plosone/s/recommended-repositories.> You also have the option of uploading the data as Supporting Information files, but we would recommend depositing data directly to a data repository if possible.

Please update your Data Availability statement in the submission form accordingly.

**Author’s response:**

The data underlying the findings of this study contain potentially identifiable and sensitive patient information derived from hospital medical records. The study was approved as an exempt retrospective study by the Institutional Review Board of Ulsan University Hospital (IRB No. UUH 2020-12-033), with a waiver of informed consent. Due to ethical and legal restrictions imposed by the Institutional Review Board and applicable privacy regulations, the data cannot be made publicly available.

Data are available from the Ulsan University Hospital Institutional Data Access Committee for researchers who meet the criteria for access to confidential data.

Requests for data access may be directed to Institutional Review Board of Ulsan University Hospital. (email: 0716782@uuh.ulsan.kr)

**Additional Editor Comments:

After considering the revised manuscript and the comments from six external referees, the majority found the work relevant, well executed, and supported by interesting results. However, I have one major concern that the authors should address. A prior publication has examined a very similar question, differing primarily in the population studied (PMID: 37517130). The authors should therefore clearly delineate how the present study differs from that work and articulate the incremental value of extending the analysis to individuals with and without CVD, rather than focusing only on diabetes.**

**Author’s response:**

Thank you for this important comment. The present study and our previous publication (PMID: 37517130) both originated from the same health screening CCTA registry. However, the two studies addressed different research questions and used distinct analytic approaches.

In the previous study, the primary objective was to investigate whether the association between hypertension and subclinical coronary atherosclerosis differed according to diabetes status. Therefore, the analysis focused on the modifying effect of diabetes mellitus, with separate analyses performed in individuals with and without diabetes. In that study, subclinical coronary atherosclerosis was primarily defined as the presence of coronary plaque on CCTA.

In contrast, the objective of the present study is different. Rather than focusing on diabetes-related effect modification, the current analysis evaluates the association between graded blood pressure categories and the severity of coronary artery disease detected by CCTA in asymptomatic individuals without prior cardiovascular disease. Specifically, we assessed imaging-based markers of disease severity, including non-obstructive CAD, obstructive CAD, and coronary artery calcium burden, across blood pressure categories. Importantly, the present study does not extend the analysis to individuals with and without established cardiovascular disease but rather focuses specifically on asymptomatic individuals without prior cardiovascular disease in order to evaluate the relationship between blood pressure categories and subclinical coronary atherosclerosis detected by CCTA.

Thus, although both studies used the same underlying registry, the clinical questions and analytical focus differ substantially. The previous study examined whether diabetes modifies the relationship between hypertension and coronary plaque, whereas the present study evaluates how different blood pressure categories are associated with the severity of subclinical coronary atherosclerosis detected by CCTA.

We believe that this complementary analysis provides additional clinical insights into the relationship between blood pressure severity and imaging-defined coronary atherosclerosis beyond the scope of the previous work.

[Note: HTML markup is below. Please do not edit.]

Reviewers' comments:

Reviewer's Responses to Questions

**Comments to the Author**

1. Is the manuscript technically sound, and do the data support the conclusions?

The manuscript must describe a technically sound piece of scientific research with data that supports the conclusions. Experiments must have been conducted rigorously, with appropriate controls, replication, and sample sizes. The conclusions must be drawn appropriately based on the data presented.

Reviewer #1: Yes

Reviewer #2: Yes

Reviewer #3: Partly

Reviewer #4: Partly

Reviewer #5: Yes

Reviewer #6: Partly

2. Has the statistical analysis been performed appropriately and rigorously?

Reviewer #1: No

Reviewer #2: Yes

Reviewer #3: Yes

Reviewer #4: Yes

Reviewer #5: Yes

Reviewer #6: Yes

3. Have the authors made all data underlying the findings in their manuscript fully available?

The [PLOS Data policy](http://track.editorialmanager.com/CL0/http:%2F%2Fwww.plosone.org%2Fstatic%2Fpolicies.action%23sharing/1/010f019c93c0c3e1-249079e6-a3ac-47a5-89e8-064ceca5b0fb-000000/Exc3ESqMh9hFZxLhgLI_r5fZpawcFMhRI3--m1YH2TU=252) requires authors to make all data underlying the findings described in their manuscript fully available without restriction, with rare exception (please refer to the Data Availability Statement in the manuscript PDF file). The data should be provided as part of the manuscript or its supporting information, or deposited to a public repository. For example, in addition to summary statistics, the data points behind means, medians and variance measures should be available. If there are restrictions on publicly sharing data—e.g. participant privacy or use of data from a third party—those must be specified.

Reviewer #1: No

Reviewer #2: Yes

Reviewer #3: No

Reviewer #4: No

Reviewer #5: No

Reviewer #6: Yes

4. Is the manuscript presented in an intelligible fashion and written in standard English?

PLOS ONE does not copyedit accepted manuscripts, so the language in submitted articles must be clear, correct, and unambiguous. Any typographical or grammatical errors should be corrected at revision, so please note any specific errors here.

Reviewer #1: Yes

Reviewer #2: Yes

Reviewer #3: Yes

Reviewer #4: Yes

Reviewer #5: Yes

Reviewer #6: Yes

5. Review Comments to the Author

Please use the space provided to explain your answers to the questions above. You may also include additional comments for the author, including concerns about dual publication, research ethics, or publication ethics. (Please upload your review as an attachment if it exceeds 20,000 characters)

**Reviewer #1: In this study Park and coworkers analyze the correlation between hypertension and extent of CAD in CCTA in a retrospective cohort. They find a correlation after correcting for other risk factors.
While the multivariate analysis is fully comprehensible, the other statistics remain vague.
All presented test for a difference between at least 2 categories out of the 4. This makes the results somehow arbitrary, a p for trend would be more meaningful, hypertension is ordinal and not categorical.
In remains unclear why two different test were used for categorical and ordinal data each.
There is date on calcium score that is not analyzed, paritcularly in this asymptomatic cohort calcium score is an important predictor. An analysis might give further insight.
Overall novelty and clinical usage of the data is limited.**

**Author’s response:**

We sincerely appreciate the reviewer’s insightful comment. In response, we performed additional analyses to evaluate the association between the degree of hypertension and coronary artery calcium (CAC) burden.

First, hypertension categories were additionally modeled as an ordinal variable to formally evaluate the linear trend across blood pressure categories. A significant linear trend was observed for both non-obstructive and obstructive CAD (**Table**).

**Table.** Test for linear trend across hypertension categories in multivariable logistic regression models

| Outcome | Linear trend OR (95% CI) | P for trend |
| --- | --- | --- |
| Non-obstructive CAD | 1.16 (1.10-1.22) | <0.001 |
| Obstructive CAD | 1.16 (1.04-1.30) | 0.008 |

Hypertension category was modeled as an ordinal variable (1 = normal, 2 = elevated, 3 = stage 1 hypertension, 4 = stage 2 hypertension)

Covariates in the multivariable model include age, sex, obesity (body mass index ≥25 kg/m^2^), diabetes mellitus, dyslipidemia, current smoking, and family history of coronary artery disease

CAD = coronary artery disease; CI = confidence interval

Second, prior studies have reported that, in comparison with coronary computed tomography angiography (CCTA) findings, a CACS of 0 is typically indicative of normal coronary arteries, whereas CACS values of 1–400 and >400 are associated with non-obstructive and obstructive CAD, respectively.

We examined the relationship between hypertension severity and coronary calcium burden using multivariable logistic regression models for CACS>100 (non-obstructive CAD) and CACS>400 (obstructive CAD) (**Table 4**). Higher BP categories were independently associated with an increased likelihood of both CACS >100 and CACS >400 after adjustment for cardiovascular risk factors. In particular, stage 2 hypertension remained significantly associated with both CACS >100 and CACS > 400, whereas stage 1 hypertension was associated with CAC > 100 but not CAC > 400. These findings demonstrate a graded relationship between blood pressure severity and coronary calcium burden. Ultimately, the CACS results were consistent with the findings from CCTA.

The corresponding results have been incorporated into the revised manuscript (**Results section 3.3**) and are presented in **Table 4.**

**Result, 3.3. Page 10**

Furthermore, the adjusted ORs for obstructive CAD were not significantly associated with stage 1 hypertension (1.261, 95% CI 0.940-1.692, p=0.122) but were significantly higher in stage 2 hypertension (1.696, 95% CI 1.194-2.409; p=0.003).

Additionally, when coronary artery calcium burden was evaluated, stage 1 hypertension was associated with CACS ≥ 1 and > 100 but not CACS > 400. On the other hand, stage 2 hypertension remained a significant association with CACS ≥ 1, > 100, and > 400 (**Table 4**).

**Table 4. Univariable and multivariable analyses for the association between degree of hypertension and coronary artery calcium score**

| Variables | Coronary artery calcium score ≥1 | | | | |
| --- | --- | --- | --- | --- | --- |
|  | Univariable analysis | | | Multivariable analysis | |
|  | Odds ratio (95% CI) | p-value | Odds ratio (95% CI) | | p-value |
| Category of blood pressure |  | <0.001 |  | | <0.001 |
| Normal (reference) | 1 | – | 1 | | – |
| Elevated | 1.466 (1.230-1.748) | <0.001 | 1.073 (0.882-1.305) | | 0.480 |
| Stage 1 | 1.949 (1.715-2.214) | <0.001 | 1.399 (1.212-1.614) | | <0.001 |
| Stage 2 | 2.371 (2.015-2.789) | <0.001 | 1.746 (1.454-2.097) | | <0.001 |
|  | Coronary artery calcium score >100 | | | |  |
| Variables | Univariable analysis | | Multivariable analysis | | |
|  | Odds ratio (95% CI) | p-value | Odds ratio (95% CI) | | p-value |
| Category of blood pressure |  | <0.001 |  | | <0.001 |
| Normal (reference) | 1 | – | 1 | | – |
| Elevated | 1.532 (1.096-2.142) | 0.013 | 1.099 (0.767-1.576) | | 0.608 |
| Stage 1 | 2.128 (1.669-2.715) | <0.001 | 1.511 (1.163-1.963) | | 0.002 |
| Stage 2 | 3.017 (2.276-3.998) | <0.001 | 2.231 (1.640-3.034) | | <0.001 |
|  | Coronary artery calcium score >400 | | | | |
| Variables | Univariable analysis | | Multivariable analysis | | |
|  | Odds ratio (95% CI) | p-value | Odds ratio (95% CI) | | p-value |
| Category of blood pressure |  | <0.001 |  | | <0.001 |
| Normal (reference) | 1 | – | 1 | | – |
| Elevated | 1.424 (0.680-2.983) | 0.349 | 0.971 (0.449-2.098) | | 0.940 |
| Stage 1 | 1.951 (1.147-3.318) | 0.014 | 1.261 (0.717-2.217) | | 0.421 |
| Stage 2 | 3.750 (2.120-6.635) | <0.001 | 2.449 (1.321-4.541) | | 0.004 |

Covariates in the multivariable model include age, sex, obesity (body mass index ≥25 kg/m^2^), diabetes mellitus, dyslipidemia, current smoking, and family history of coronary artery disease

CI = conﬁdence interval.

**References**

1. Rumberger JA, Brundage BH, Rader DJ, et al. Electron beam computed tomographic coronary calcium scanning: a review and guidelines for use in asymptomatic persons. Mayo Clin Proc. 1999;74:243-52.

**Reviewer #2: The authors retrospectively investigated the degree of hypertension and non-obstructive and obstructive coronary disease in 7332 asymptomatic subjects without cardiovascular disease who underwent CCTA for a general health examination (mean age 52.8 ± 7.8 years, 63.8% male). Hypertension was graded as normal (<120/80 mmHg), elevated (120-129/ <80 mmHg), stage 1 hypertension (130-139/80-89 mmHg), and stage 2 hypertension (≥140/90 mmHg) according to ACC/AHA 2025 guidelines. Coronary artery calcium score (CACS) was classified as 0, 1-10, 11-100, 101-400, and >400, and stenosis ≥50% on CCTA was defined as obstructive CAD. The CACS grade and mean value of CACS were increased progressively with increasing hypertension stages. The degree of hypertension was also associated with the prevalence of obstructive CAD and non-obstructive CAD, and the stage 2 hypertension group showed a higher prevalence of both types of CAD compared with the other groups. Multivariate logistic regression analysis indicated that non-obstructive CAD was significantly associated with both stage 1 and stage 2 hypertension after adjustment for other risk factors, while obstructive CAD was associated with stage 2 hypertension, but not with stage 1 hypertension.

1) The major finding of the present study is that higher stages of hypertension are associated with higher CACS even in asymptomatic subjects. Similar findings were previously reported in several studies (e.g., Kang J, Atherosclerosis. 2019;282:188-195, Im TS, Int J Cardiovasc Imaging. 2014;30 Suppl 2:105-112, etc.). The authors should indicate the differences between this study and previous studies in the Discussion section. In particular, the authors should emphasize how their application of the new hypertension grading, according to the new ACC/AHA guidelines, distinguishes their findings from previous research.**

**Author’s response:**

We thank the reviewer for this thoughtful comment.

We agree that previous studies, including those by Im et al. and Kang et al. have demonstrated a graded association between blood pressure and coronary artery calcium burden. In particular, Im et al. reported increasing adjusted odds ratios for subclinical coronary atherosclerosis and CACS > 100 across JNC 7 blood pressure categories, while Kang et al. showed a stepwise association between ACC/AHA hypertension stages and the prevalence of coronary artery calcium. Consistent with these prior findings, our results also demonstrate a graded relationship between increasing hypertension severity and coronary calcium burden.

However, several important differences distinguish our study from previous investigations. First, earlier CCTA-based studies such as that by Im et al., applied the JNC 7 classification system, in which individuals with BP 120-139/80-89 mmHg were categorized as “prehypertension.” This classification differs from the BP categorization used in our study, which follows the contemporary 2025 ACC/AHA guidelines. Because the BP categories and their ranges differ between the two studies, direct comparisons of results across specific BP groups are inherently limited. Nevertheless, both studies show a similar overall pattern in which increasing BP severity is associated with greater coronary atherosclerotic burden.

Second, although Kang et al. applied the 2017 ACC/AHA classification, whereas our study used the 2025 ACC/AHA guideline, the numerical blood pressure thresholds were essentially the same. However, their analysis primarily evaluated the presence of coronary artery calcium (CACS>0), while our study assessed CAC burden in greater detail (CACS>1, > 100, and > 400) and performed regression analyses according to hypertension stage. In addition, using CCTA, we evaluated both coronary calcium and luminal stenosis severity, demonstrating that stage 1 hypertension was independently associated with non-obstructive CAD, whereas stage 2 hypertension was associated with both non-obstructive and obstructive CAD.

Taken together, our findings extend previous observations by providing a more detailed assessment of coronary calcium burden and coronary artery stenosis severity across contemporary hypertension stages. We have revised the **Discussion** section accordingly as follow:

**Discussion, Page 11**

In addition to stenosis-based CAD severity, we observed a graded increase in coronary artery calcium burden across hypertension stages, including higher odds of CACS ≥ 1, > 100, and > 400 in multivariable analyses. Coronary artery calcium is a well-established marker of subclinical atherosclerosis and an important predictor of future cardiovascular risk [26]. Previous studies in asymptomatic populations have similarly reported higher coronary calcium burden with increasing blood pressure severity, although differences exist in BP classification systems and analytic approaches [27, 28]. Despite these differences, the overall trend is consistent, supporting the concept that greater blood pressure severity is associated with a coronary calcium burden reflecting total atherosclerotic burden.

**2) What were the reasons for performing CCTA in 10,581 asymptomatic patients? Please indicate the major reasons for CCTA in this study cohort.**

**Author’s response:**

We thank the reviewer for this important comment.

As a compulsory social insurance, South Korea has a National Health Insurance (NHI) system which covers the whole population living in the country. NHI has actively operated the business of promoting health checkups and health level in an effort to detect diseases early and enhance public health (**Figure 1**). These general health screenings have been performed to the employee insured with no age limit (annually) and the self-employed insured such as householders or dependents older than 40 year-old (biannually). These general checkups are free and covered by NIH. Anyone can receive general medical checkups at any hospital in the country. These general heath checkups include past and current medical history including smoking and alcohol status, exercise status, family medical history, physical examination, height, weight, waist circumference, systolic and diastolic blood pressure, blood tests (fasting blood glucose, fasting total cholesterol, high-density lipoprotein cholesterol, low-density lipoprotein cholesterol, triglycerides, hemoglobin, creatinine, aspartate transaminase [AST], alanine transferase,[ALT], gamma glutamyl transferase), urinalysis, and chest X-ray. All general heath checkups are reviewed and examined by doctors.

The present study included individuals who had these general medical checkups. Since Ulsan University Hospital is the only Tertiary Hospital in Ulsan Metropolitan City, this hospital holds a lot of health information of individuals who had voluntarily undergone a general health examination. In addition, if individuals undergoing general medical checkups paid additional costs, they could take additional blood or imaging tests such as coronary computed tomography angiography (CCTA), abdominal ultrasonography, brain MRI and so on.

In the present study, the study population consisted of 10,581 consecutive asymptomatic individuals who voluntarily underwent self-referred CCTA as part of a general health examination at the Health Promotion Center of Ulsan University Hospital. At the time of the examination, the potential risks associated with CCTA, including radiation exposure and contrast use, were explained to all participants.

Therefore, the primary reason for performing CCTA in this cohort was voluntary screening for the early detection of subclinical coronary artery disease within a health examination program, rather than symptom-driven or physician-directed diagnostic evaluation.

**Figure 1.** NHI Health promotion system in South Korea

**References**

1. National Health Insurance Service. https://www.nhis.or.kr/english/index.do

**3) Although all continuous values are presented as mean ± standard deviation, those without normal distribution should be presented as median (interquartile range) for accurate representation.**

**Author’s response:**

We thank the reviewer for this important comment.

In response, we assessed the normality of continuous variables using the Shapiro-Wilk test. Variables that did not follow a normal distribution (fasting blood glucose, hemoglobin A1c, triglycerides, ASCVD risk score, mean coronary artery calcium score) are now presented as median (interquartile range) in **Table 1 and 2**. Accordingly, the Statistical Analysis section (**Methods 2.5**) has been updated to clarify that continuous variables are presented as mean ± standard deviation or median (interquartile range), as appropriate, and that between-group comparisons were performed using one-way analysis of variance or the Kruskal-Wallis test, as appropriate.

**Methods, 2.5. Statistical analysis, Page 8**

Categorical variables are presented as frequencies and percentages. Continuous variables are presented as mean ± standard deviation or median (interquartile range), as appropriate. Pearson’s chi-square test or Fisher’s exact test was used to compare categorical variables, whereas continuous variables were evaluated using one-way analysis of variance or Kruskal-Wallis test, as appropriate for between-group comparisons.

**Table 1. Baseline characteristics of the study population according to the degree of hypertension**

| Characteristics | The category of blood pressure | | | | | |
| --- | --- | --- | --- | --- | --- | --- |
|  | Overall  (n = 7,332) | Normal  (n = 2,500) | Elevated  (n = 969) | Stage 1 Hypertension  (n = 2,841) | Stage 2 Hypertension  (n = 1,022) | p-value |
| Age, years | 52.8 ± 7.8 | 51.9 ± 7.5 | 53.8 ± 7.7 | 53.1 ± 7.8 | 53.7 ± 8.4 | <0.001 |
| Men, no. (%) | 4,680 (63.8) | 1,276 (51.0) | 608 (62.7) | 2,092 (73.6) | 704 (68.9) | <0.001 |
| Systolic blood pressure, mmHg | 123.8 ± 13.8 | 109.7 ± 7.2 | 123.9 ± 2.9 | 128.6 ± 7.0 | 144.8 ± 10.6 | <0.001 |
| Diastolic blood pressure, mmHg | 78.0 ± 9.5 | 69.7 ± 6.1 | 73.7 ± 4.6 | 82.1 ± 4.5 | 91.4 ± 8.2 | <0.001 |
| Body mass index, kg/m^2^ | 23.9 ± 2.9 | 22.9 ± 2.6 | 24.0 ± 2.6 | 24.4 ± 2.7 | 25.2 ± 3.2 | <0.001 |
| Waist circumference, cm | 84.9 ± 7.6 | 82.1 ± 7.1 | 85.2 ± 7.1 | 86.1 ± 7.2 | 87.9 ± 8.2 | <0.001 |
| Diabetes mellitus, no. (%) | 750 (10.2) | 189 (7.6) | 100 (10.3) | 324 (11.4) | 137 (13.4) | <0.001 |
| Dyslipidemia, no. (%) | 1,285 (17.5) | 374 (15.0) | 201 (20.7) | 522 (18.4) | 188 (18.4) | 0.001 |
| Current smoker, no. (%) | 1,647 (22.9) | 531 (21.6) | 207 (21.8) | 696 (24.9) | 213 (21.2) | 0.010 |
| Obesity, no. (%) | 2,392 (32.8) | 497 (19.9) | 309 (32.2) | 1,090 (38.5) | 496 (48.9) | <0.001 |
| Family history of coronary artery disease^*^ | 660 (9.0) | 212 (8.5) | 93 (9.6) | 258 (9.1) | 97 (9.5) | 0.673 |
| Fasting blood glucose, mg/dL | 91 (84-99) | 89 (82-96) | 91 (84-99) | 92 (85-101) | 94 (85.5-104) | <0.001 |
| Hemoglobin A1c, % | 5.5 (5.3-5.8) | 5.4 (5.2-5.7) | 5.5 (5.3-5.8) | 5.5 (5.3-5.8) | 5.6 (5.3-5.9) | <0.001 |
| Total cholesterol, mg/dL | 193.8 ± 36.5 | 192.2 ± 34.7 | 193.6 ± 39.3 | 194.2 ± 36.7 | 197.0 ± 37.4 | 0.012 |
| LDL cholesterol, mg/dL | 129.8 ± 34.1 | 126.6 ± 32.4 | 131.9 ± 36.5 | 130.5 ± 34.0 | 133.7 ± 34.9 | <0.001 |
| HDL cholesterol, mg/dL | 53.4 ± 14.9 | 55.4 ± 15.1 | 52.8 ± 14.5 | 52.4 ± 15.0 | 51.8 ± 14.4 | <0.001 |
| Triglyceride, mg/dL | 94 (66-138) | 85 (60-121) | 94 (66-134) | 102 (71-149) | 105 (71-152) | <0.001 |
| Creatinine, mg/dL | 0.84 ± 0.18 | 0.82 ± 0.18 | 0.83 ± 0.18 | 0.87 ± 0.17 | 0.85 ± 0.19 | <0.001 |
| Uric acid, mg/dL | 5.33 ± 1.34 | 5.05 ± 1.28 | 5.27 ± 1.29 | 5.49 ± 1.34 | 5.63 ± 1.40 | <0.001 |
| Ejection fraction, % | 64.3 ± 4.5 | 64.5 ± 4.3 | 64.0 ± 5.6 | 64.3 ± 4.2 | 64.0 ± 4.9 | 0.048 |
| ASCVD risk score | 3.9 (1.6-7.7) | 2.2 (0.9-5.0) | 4.1 (1.9-7.9) | 4.9 (2.3-8.7) | 5.6 (3.0-11.0) | <0.001 |

Values are shown as mean ± standard deviation, median (interquartile range), or number (%), as appropriate.

^*^Coronary artery disease in a ﬁrst-degree relative of any age.

ASCVD = atherosclerotic cardiovascular disease; HDL = high density lipoprotein; LDL = low density lipoprotein

**Table 2. Comparison of coronary computed tomography angiographic findings according to the degree of hypertension**

| Variables | The category of blood pressure | | | | | |
| --- | --- | --- | --- | --- | --- | --- |
|  | Overall  (n = 7,332) | Normal  (n = 2,500) | Elevated  (n = 969) | Stage 1 Hypertension  (n = 2,841) | Stage 2 Hypertension  (n = 1,022) | p-value |
| Coronary artery calcium score | 0 (0-1.6) | 0 (0) | 0 (0-1.0) | 0 (0-5.5) | 0 (0-12.0) | <0.001 |

Values are shown as mean ± standard deviation, median (interquartile range), or number (%), as appropriate.

**4) Patients with a CACS of 0 have an extremely low risk of major adverse cardiovascular events (MACE) and all-cause mortality, with event rates consistently <1% per year. Those with a CAC score ≥1 have a significantly higher risk of cardiovascular events, with risk increasing as the score rises. Even minimal CACS (1-99) confers a borderline to intermediate 10-year risk (5-7.5%). Thus, it is clinically important to differentiate those with a CACS 0f 0 from those with CACS with ≥1. The authors should investigate the association between CACS=0 and hypertension grade.**

**Author’s response:**

We thank the reviewer for this important and clinically relevant comment. We agree that distinguishing individuals with CACS =0 from those with any detectable coronary calcium (CACS ≥1) is meaningful for cardiovascular risk stratification.

Previous studies have reported that, in comparison with coronary computed tomography angiography (CCTA) findings, a CACS of 0 is typically indicative of normal coronary arteries, whereas CACS values of 1–400 and >400 are associated with non-obstructive and obstructive CAD, respectively.

Accordingly, we performed an additional multivariable logistic regression analysis comparing participants with CACS = 0 versus CACS ≥1. Specifically, stage 1 hypertension and stage 2 hypertension were significantly associated with CACS ≥1, whereas elevated BP was not independently associated after adjustment. These findings suggest that increasing hypertension severity is associated with the presence of any detectable coronary calcium (CACS ≥1). Consequently, the CACS results were consistent with the findings from CCTA.

The corresponding results have been incorporated into the revised manuscript (**Results section 3.3**) and are presented in **Table 4.**

**Result, 3.3. Page 10**

Furthermore, the adjusted ORs for obstructive CAD were not significantly associated with stage 1 hypertension (1.261, 95% CI 0.940-1.692, p=0.122) but were significantly higher in stage 2 hypertension (1.696, 95% CI 1.194-2.409; p=0.003).

Additionally, when coronary artery calcium burden was evaluated, stage 1 hypertension was associated with CACS ≥ 1 and > 100 but not CACS > 400. On the other hand, stage 2 hypertension remained a significant association with CACS ≥ 1, > 100, and > 400 (**Table 4**).

**Table 4. Univariable and multivariable analyses for the association between degree of hypertension and coronary artery calcium score**

| Variables | Coronary artery calcium score ≥1 | | | | |
| --- | --- | --- | --- | --- | --- |
|  | Univariable analysis | | | Multivariable analysis | |
|  | Odds ratio (95% CI) | p-value | Odds ratio (95% CI) | | p-value |
| Category of blood pressure |  | <0.001 |  | | <0.001 |
| Normal (reference) | 1 | – | 1 | | – |
| Elevated | 1.466 (1.230-1.748) | <0.001 | 1.073 (0.882-1.305) | | 0.480 |
| Stage 1 | 1.949 (1.715-2.214) | <0.001 | 1.399 (1.212-1.614) | | <0.001 |
| Stage 2 | 2.371 (2.015-2.789) | <0.001 | 1.746 (1.454-2.097) | | <0.001 |
|  | Coronary artery calcium score >100 | | | |  |
| Variables | Univariable analysis | | Multivariable analysis | | |
|  | Odds ratio (95% CI) | p-value | Odds ratio (95% CI) | | p-value |
| Category of blood pressure |  | <0.001 |  | | <0.001 |
| Normal (reference) | 1 | – | 1 | | – |
| Elevated | 1.532 (1.096-2.142) | 0.013 | 1.099 (0.767-1.576) | | 0.608 |
| Stage 1 | 2.128 (1.669-2.715) | <0.001 | 1.511 (1.163-1.963) | | 0.002 |
| Stage 2 | 3.017 (2.276-3.998) | <0.001 | 2.231 (1.640-3.034) | | <0.001 |
|  | Coronary artery calcium score >400 | | | | |
| Variables | Univariable analysis | | Multivariable analysis | | |
|  | Odds ratio (95% CI) | p-value | Odds ratio (95% CI) | | p-value |
| Category of blood pressure |  | <0.001 |  | | <0.001 |
| Normal (reference) | 1 | – | 1 | | – |
| Elevated | 1.424 (0.680-2.983) | 0.349 | 0.971 (0.449-2.098) | | 0.940 |
| Stage 1 | 1.951 (1.147-3.318) | 0.014 | 1.261 (0.717-2.217) | | 0.421 |
| Stage 2 | 3.750 (2.120-6.635) | <0.001 | 2.449 (1.321-4.541) | | 0.004 |

Covariates in the multivariable model include age, sex, obesity (body mass index ≥25 kg/m^2^), diabetes mellitus, dyslipidemia, current smoking, and family history of coronary artery disease

CI = conﬁdence interval.

**References**

1. Rumberger JA, Brundage BH, Rader DJ, et al. Electron beam computed tomographic coronary calcium scanning: a review and guidelines for use in asymptomatic persons. Mayo Clin Proc. 1999;74:243-52.

**5) There is a difference among the groups based on hypertension stages in several clinical factors (age, sex, BMI, etc.) and comorbidities. I recommend performing subgroup analyses according to these factors to explore whether they modify the association between hypertension grade and CACS or coronary stenosis.**

**Author’s response:**

We thank the reviewer for this valuable suggestion.

To address this comment, we performed additional interaction analyses to evaluate whether the association between hypertension stage and CAD outcomes differed according to key clinical factors, including sex, age group, and obesity.

For non-obstructive CAD, a significant interaction between hypertension stage and age group was observed (p for interaction = 0.001). In contrast, no significant interactions were observed for sex or obesity in relation to non-obstructive CAD. For obstructive CAD, no significant interactions were identified for age group, sex or obesity (**Table**).

As these analyses were exploratory and not central to the primary objective of the present study, the detailed results are provided below for the reviewer’s reference and were not included in the main manuscript. We hope that the reviewer will generously understand this. Further studies with larger populations and longitudinal designs are needed to better clarify the potential modifying effects of these clinical factors.

**Table. Interaction analyses evaluating whether clinical factors modify the association between hypertension stage and CAD outcomes.**

| Outcome | Effect modifier | p for interaction |
| --- | --- | --- |
| Non-obstructive CAD | Sex | 0.385 |
|  | Obesity | 0.439 |
|  | age group* | 0.001 |
| Obstructive CAD | Sex | 0.913 |
|  | Obesity | 0.248 |
|  | age group* | 0.636 |

* Age group was categorized as < 70 years and ≥ 70 years.

P values represent the interaction terms obtained from multivariable logistic regression models adjusting for age, sex, obesity, diabetes, dyslipidemia, current smoking, and family history of CAD.

**6) Subjects of this study were divided into 5 groups as CACS 0, 1–10, 11–100, 101–400, >400. Although this stratification is acceptable, it may be helpful to additionally perform analyses using more commonly employed CACS categories (0, 1–99, 100–299, ≥300), which are frequently used in recent literature (see Slipczuk L, Blankstein R, Bucciarelli-Ducci C, et al. Circulation. 2025;152:e443-e466), to facilitate comparison with other studies.**

**Author’s response:**

We thank the reviewer for this important comment. As suggested, we performed an additional analysis using alternative coronary artery calcium score (CACS) categories (0, 1-99, 100-299, ≥300) (**Table**). Using these revised categories, the distribution of CACS across blood pressure groups demonstrated a consistent graded association. The proportion of individuals with higher CACS categories (100-299 and ≥300) progressively increased with increasing blood pressure severity (p<0.001). Importantly, the overall pattern and statistical significance remained unchanged compared with our original categorization. Given that these additional analyses yielded highly consistent results and did not significantly alter the interpretation of our findings, we have opted to retain the original categorization in the main manuscript to preserve the analytic framework and internal consistency of the study. We hope that the reviewer will generously understand this. The results using the alternative CACS categories are provided here for the reviewer’s consideration.

**Table. Distribution of coronary artery calcium score categories according to blood pressure classification**

| Variables | The category of blood pressure | | | | | |
| --- | --- | --- | --- | --- | --- | --- |
|  | Overall  (n = 7,332) | Normal  (n = 2,500) | Elevated  (n = 969) | Stage 1 HTN  (n = 2,841) | Stage 2 HTN  (n = 1,022) | p-value |
| CACS, no. (%) |  |  |  |  |  | <0.001 |
| 0 | 5356 (73.1%) | 2025 (81.1%) | 722 (74.5%) | 1951 (68.7%) | 658 (64.4%) |  |
| 1-99 | 1475 (20.1%) | 374 (15.0%) | 190 (19.6%) | 659 (23.2%) | 245 (24.7%) |  |
| 100-299 | 333 (4.5%) | 70 (2.8%) | 36 (3.7%) | 155 (5.5%) | 72 (7.0%) |  |
| ≥300 | 164 (2.2%) | 29 (1.2%) | 21 (2.2%) | 74 (2.6%) | 40 (3.9%) |  |

**Minor comments:
“Dyslipidemia” is a more suitable term than “hyperlipidemia”.**

**Author’s response:**

We thank the reviewer for this helpful suggestion. The term “hyperlipidemia” has been replaced with “dyslipidemia throughout the manuscript for greater accuracy and consistency with current terminology.

**Reviewer #3: Manuscript: Degree of hypertension and subclinical coronary atherosclerosis in asymptomatic individuals without cardiovascular disease

General Assessment
This manuscript presents a large retrospective cross-sectional analysis of 7,332 asymptomatic individuals undergoing coronary computed tomography angiography (CCTA) as part of a health screening program. The authors investigate the association between blood pressure (BP) categories defined by contemporary ACC/AHA thresholds and the presence and severity of subclinical coronary artery disease (CAD).
The dataset is substantial, the imaging methodology appears appropriate, and the statistical analyses are coherent and generally robust. The findings demonstrate statistically significant graded associations between higher BP categories and both non-obstructive and obstructive CAD, consistent with established pathophysiologic expectations.
However, several important methodological and interpretative aspects require clarification to ensure that the manuscript’s conclusions are fully supported and appropriately framed. The overall direction of the findings is consistent with existing evidence, and the primary issues relate to framing and proportional interpretation rather than analytic validity.

1) Main Claims and Their Significance
The central claim of the manuscript is that an increase in BP category is associated with a greater prevalence and severity of subclinical CAD detected by CCTA in asymptomatic individuals without known cardiovascular disease.
This is a clinically and epidemiologically meaningful question. However, the manuscript currently presents the association as if it addresses an unresolved uncertainty in the field. In reality, the relationship between elevated BP and coronary atherosclerosis is well established epidemiologically. The study does not represent a paradigm shift, but rather provides large-scale imaging-based confirmation within a screening cohort using contemporary BP thresholds. In this context, the contribution of the present study is primarily incremental rather than transformative, which is entirely appropriate provided that it is positioned with precision.
To strengthen the manuscript, clarification is needed regarding the specific gap this study addresses. In particular, the manuscript would benefit from a more apparent distinction between confirmatory evidence and novel contribution, and from explicitly defining whether the added value lies in the screening population, the untreated BP phenotype, or the imaging-based assessment.
This refinement would enhance intellectual coherence without requiring additional data.**

**Author’s response:**

Thank you for this thoughtful comment. We agree with the reviewer that the epidemiological relationship between elevated blood pressure and coronary atherosclerosis has been well established in prior studies. The intention of the present study was therefore not to suggest a paradigm shift, but rather to provide additional imaging-based evidence of this association with an asymptomatic screening population using contemporary blood pressure categories.

In response to the reviewer’s suggestion, we have revised the **Introduction** to more clearly define the specific gap addressed by this study. In particular, we now emphasize that although the association between blood pressure and cardiovascular risk has been well established epidemiologically, large-scale imaging evidence evaluating subclinical coronary atherosclerosis across contemporary BP categories in asymptomatic screening populations remains limited.

In addition, we reviewed the **Discussion** to ensure that the findings are consistently framed as associations observed in a cross-sectional imaging study and are interpreted in the context of previously established epidemiological evidence. Specifically, we added a sentence noting that our findings are consistent with prior epidemiological studies and extend this evidence through imaging-based assessment in an asymptomatic screening population.

We believe these revisions clarify the positioning of the present study and better distinguish the contribution of the imaging-based assessment within a large screening cohort.

**Introduction, Page 5**

However, large-scale imaging evidence evaluating subclinical coronary atherosclerosis across contemporary BP categories in asymptomatic screening populations remains limited. Therefore, in this study, we aimed to evaluate the association between the degree of hypertension and the presence and severity of subclinical coronary atherosclerosis in a large cohort of asymptomatic individuals who underwent CCTA during a health check-up.

**Discussion, Page 10, 11**

Both stage 1 and stage 2 hypertension were independently associated with non-obstructive CAD, while stage 2 hypertension was additionally associated with obstructive CAD. These findings are consistent with prior epidemiological studies demonstrating a graded association between blood pressure and coronary atherosclerosis and extend this evidence through imaging-based assessment in an asymptomatic screening population [11, 25].

**2) Definition of Exposure: Blood Pressure Phenotype vs Clinical Hypertension
A central conceptual issue concerns how “hypertension” is defined. BP categories are derived from measurements obtained at a single visit, using the average of the second and third readings. Additionally, individuals receiving antihypertensive therapy were excluded (as shown in the flow diagram). Therefore, the study does not evaluate clinically diagnosed hypertension as a chronic condition. Instead, it examines a blood pressure phenotype measured at a single encounter in untreated individuals. This distinction is critical, as clinical diagnosis of hypertension is inherently longitudinal and requires repeated measurements. Single-visit BP classification may lead to nondifferential misclassification. Excluding treated individuals removes a biologically and clinically important segment of the hypertensive population.
At the same time, this design creates a unique opportunity: it isolates the association between current hemodynamic exposure and coronary morphology, free from pharmacologic modification.
The manuscript would benefit from explicitly clarifying in the Methods that BP classification is based on single-visit measurement. Potential misclassification should be acknowledged in the Discussion, and language implying conclusions about diagnosed hypertension may need to be reconsidered. In addition, the implications of excluding treated individuals deserve further discussion, particularly with respect to biological interpretation and generalizability. This clarification would significantly improve conceptual precision.**

**Author’s response:**

We thank the Reviewer for this important comment. In the present study, blood pressure was measured during a single health screening visit. To enhance measurement reliability, systolic and diastolic blood pressures were recorded three times, and the average of the second and third measurements was used for classification. Accordingly, hypertension categories were defined based on standardized office blood pressure measurements obtained at a single visit. We acknowledge that single-visit office blood pressure measurements may not fully reflect an individual’s usual blood pressure level and may introduce nondifferential misclassification, including the possibility of white coat or masked hypertension. In response to reviewer’s suggestion, we have revised the **Limitations section**.

With respect to the reviewer’s comment regarding the exclusion of individuals receiving antihypertensive therapy, these participants were excluded to avoid potential pharmacologic modification of measured blood pressure levels, allowing evaluation of the association between current hemodynamic exposure and coronary morphology. This exclusion criterion has been clarified in the Methods section. Because antihypertensive medication use was already part of the study population definition, we did not expand the discussion of this point in the manuscript in order to maintain the overall focus and flow of the Discussion.

We believe that these revisions improve conceptual clarity and transparency.

**Discussion, Page 13**

Sixth, blood pressure was assessed during a single health screening visit. Although systolic and diastolic blood pressures were measured three times and the average of the second and third readings was used to improve reliability, single-visit measurements may not fully reflect usual blood pressure levels. Furthermore, out-of-office measurements such as home blood pressure or ambulatory blood pressure monitoring were not available. Therefore, the possibility of white coat hypertension or masked hypertension cannot be excluded.

**3) Placement Within Existing Literature
The Discussion heavily references prospective and interventional trials (e.g., BP-lowering trials and prognostic imaging studies). While such references provide context, the present study is cross-sectional and observational. Data in the manuscript demonstrate association, not causation. They do not evaluate treatment response or long-term outcomes. Accordingly, a more precise separation between cross-sectional findings and interventional evidence is warranted. In particular, the manuscript should avoid implying that the present data directly support specific therapeutic strategies and instead frame conclusions at the level of vascular phenotype and subclinical remodelling rather than at the level of intervention. This will ensure proportionality between design and interpretation.**

**Author’s response:**

Thank you for this important comment. We agree that the present study is cross-sectional and observational in nature and therefore demonstrates associations rather than causal relationships or treatment effects, as noted in the **limitations section**.

In response to the reviewer’s suggestion, we have revised several sentences in the **Abstract,** **Discussion and Conclusion sections** to avoid implying that the present data directly support specific therapeutic strategies.

**Abstract, Conclusions, page 3**

In this large cross-sectional study, the degree of hypertension was associated with an increased risk and severity of subclinical coronary atherosclerosis. These findings highlight the potential importance of early recognition of blood pressure elevation and cardiovascular risk stratification in asymptomatic individuals.

**Discussion, page 12**

Furthermore, long-term follow-up studies have shown that patients with non-obstructive CAD detected by CCTA are associated with an approximately two- to three-fold higher risk of all-cause mortality, nonfatal myocardial infarction, and subsequent revascularization [6, 33-36]. Our findings suggest that early hypertension may already be associated with subclinical coronary atherosclerosis and that a personalized approach is needed to reduce future coronary events in such early hypertensive individuals.

**Discussion, page 12**

This suggests that subclinical atherosclerosis may already be present even in patients traditionally classified as lower risk~~, underscoring potential gaps in existing recommendations and highlighting the need for more tailored preventive strategies in this population~~.

**Discussion, page 13**

Furthermore, in a large 10-year follow-up study, CCTA assessed CAD severity (normal, nonobstructive, or obstructive) and provided robust prognostic power for predicting cardiac death and nonfatal MI, clearly delineating diverging long-term risk trajectories and substantially improving risk reclassification [37]. Taken together, our findings suggest that increasing hypertension severity is associated with a greater burden of coronary atherosclerosis, highlighting the importance of early recognition of elevated blood pressure.

**Conclusion, page 14**

In conclusion, the degree of hypertension was significantly associated with the presence and severity of subclinical coronary atherosclerosis in asymptomatic individuals. These findings suggest that even early hypertension may be associated with subclinical coronary atherosclerosis, highlighting the importance of early recognition of blood pressure elevation and appropriate cardiovascular risk stratification.

**4) Effect Size and Clinical Interpretation
The adjusted odds ratios reported in the manuscript (approximately 1.3–1.7) indicate statistically significant associations between higher BP categories and the presence of non-obstructive and obstructive CAD. However, the clinical significance of these associations cannot be fully appreciated from odds ratios alone. To enhance interpretability, presenting absolute CAD prevalence across BP categories would be valuable. Reporting adjusted predicted probabilities derived from the multivariable model may further translate odds ratios into clinically interpretable estimates. Finally, statements implying substantial risk increase should be proportionate to the observed magnitude of association.
Providing absolute and adjusted risk estimates would substantially enhance clinical interpretability. This distinction is particularly relevant in large cohorts, where statistical significance may not fully reflect clinical magnitude.**

**Author’s response:**

We thank the Reviewer for this valuable comment. In addition to the absolute prevalence reported in **Table 2**, we calculated adjusted predicted probabilities derived from the multivariable logistic regression model to further aid clinical interpretation.

For non-obstructive CAD, the adjusted predicted probability increased progressively across blood pressure categories: 17.4% in the normal BP group, 21.3% in the elevated BP group, 27.7% in stage 1 hypertension, and 29.7% in stage 2 hypertension. Similarly, for obstructive CAD, the adjusted predicted probability increased from 3.1% in the normal BP group to 5.3% in the elevated BP group, 5.4% in stage 1 hypertension, and 7.0% in stage 2 hypertension.

These adjusted estimates closely align with the crude prevalence presented in **Table 2** and demonstrate a consistent association between hypertension severity and subclinical coronary atherosclerosis. As these additional estimates do not substantially alter the interpretation of our findings, we have opted not to incorporate them into the main manuscript but provide them here for the Reviewer’s consideration. We hope that the reviewer will generously understand this.

**Table 2. Comparison of coronary computed tomography angiographic findings according to the degree of hypertension**

| Variables | The category of blood pressure | | | | | |
| --- | --- | --- | --- | --- | --- | --- |
|  | Overall  (n = 7,332) | Normal  (n = 2,500) | Elevated  (n = 969) | Stage 1 Hypertension  (n = 2,841) | Stage 2 Hypertension  (n = 1,022) | p-value |
| Mean coronary artery calcium score | 0 (0-1.6) | 0 (0) | 0 (0-1.0) | 0 (0-5.5) | 0 (0-12.0) | <0.001 |
| Coronary artery calcium score, no. (%) |  |  |  |  |  | <0.001 |
| 0 | 5,356 (73.1) | 2,025 (81.1) | 722 (74.5) | 1,951 (68.7) | 658 (64.4) |  |
| 1-10 | 587 (8.0) | 146 (5.8) | 72 (7.4) | 275 (9.7) | 94 (9.2) |  |
| 11-100 | 892 (12.2) | 230 (9.2) | 118 (12.2) | 386 (13.6) | 158 (15.5) |  |
| 101-400 | 388 (5.3) | 77 (3.1) | 46 (4.7) | 183 (6.4) | 82 (8.0) |  |
| >400 | 105 (1.4) | 20 (0.8) | 11 (1.1) | 44 (1.5) | 30 (2.9) |  |
| Severity of subclinical coronary atherosclerosis | |  |  |  |  | <0.001 |
| Normal | 5,235 (71.4) | 1,984 (79.4) | 708 (73.1) | 1,900 (66.9) | 643 (62.9) |  |
| Non-obstructive CAD | 1,743 (23.8) | 437 (17.5) | 211 (21.8) | 787 (27.7) | 308 (30.1) |  |
| Obstructive CAD | 354 (4.8) | 79 (3.2) | 50 (5.2) | 154 (5.4) | 71 (6.9) |  |

Values are shown as mean ± standard deviation or number (%).

CAD = coronary artery disease

**5) Statistical Modelling Transparency
While the multivariable logistic regression models appear appropriate, the manuscript does not provide sufficient detail regarding model diagnostics and variable selection strategy. Greater transparency regarding statistical modelling would strengthen the manuscript. It would be helpful to indicate whether multicollinearity diagnostics (e.g., Variance Inflation Factor) were performed, to clarify the rationale for covariate selection, and to specify the statistical tests underlying the comparisons in Table 1. Given the ordinal structure of CAD severity, a brief justification for the choice of separate binary models would also be informative.
These clarifications would strengthen the methodological rigour of the manuscript without requiring substantial reanalysis.**

**Author’s response:**

We thank the Reviewer for this valuable comment regarding multivariable model specification.

First, covariates were selected a priori based on clinical relevance and established cardiovascular risk factors. Specifically, age, sex, obesity, diabetes mellitus, dyslipidemia, current smoking, and family history of CAD were included as potential confounders based on their well-established associations with coronary artery disease [1]. Furthermore, we assessed multicollinearity among covariates using variance inflation factors (VIFs), and no evidence of problematic multicollinearity was observed (all VIFs < 2.0; **Table 1**).

Second, the statistical test used for comparisons in Table 1 has been clarified on **Method; 2.5 Statistical analysis section**. Categorical variables were compared using Pearson’s chi-square test or Fisher’s exact test, and continuous variables were compared using one-way ANOVA or the Kruskal-Wallis test, as appropriate.

Finally, although CAD severity can be viewed as an ordinal outcome, we used separate binary logistic regression models to distinguish clinically meaningful endpoints (non-obstructive CAD and obstructive CAD) independently. Non-obstructive CAD is increasingly recognized as a substantial at-risk state associated with adverse cardiovascular outcomes, whereas obstructive CAD is a well-established determinant of prognosis and major cardiovascular events [2, 3]. Given these distinct prognostic implications, we considered this approach clinically meaningful to evaluate the associations with each condition separately. This approach allows clearer interpretation of the relationship between hypertension severity and each clinically relevant stage of coronary atherosclerosis.

**Table 1. Variance inflation factors (VIFs) for covariates included in the multivariable regression model.**

| **Covariate** | **Collinearity Statistics** | |
| --- | --- | --- |
|  | **Tolerance** | **VIF** |
| Age | 0.958 | 1.043 |
| Sex | 0.848 | 1.179 |
| Obesity (BMI ≥ 25 kg/m^2)_^ | 0.968 | 1.033 |
| Diabetes mellitus | 0.975 | 1.026 |
| Dyslipidemia | 0.997 | 1.003 |
| Current smoking | 0.853 | 1.173 |
| Family history of CAD | 0.996 | 1.004 |

**Reference**

1. Goff DC Jr, Lloyd-Jones DM, Bennett G, et al. 2013 ACC/AHA guideline on the assessment of cardiovascular risk: a report of the American College of Cardiology/American Heart Association Task Force on Practice Guidelines. J Am Coll Cardiol. 2014 Jul 1;63(25 Pt B):2935-2959.
2. Lin FY, Shaw LJ, Dunning AM et al (2011) Mortality risk in symptomatic patients with nonobstructive coronary artery disease: a prospective 2-center study of 2,583 patients undergoing 64-detector row coronary computed tomographic angiography. J Am Coll Cardiol 58:510-519. <https://doi.org/10.1016/j.jacc.2010.11.078>
3. Bittencourt MS, Hulten E, Ghoshhajra B et al (2014) Prognostic value of nonobstructive and obstructive coronary artery disease detected by coronary computed tomography angiography to identify cardiovascular events. Circ Cardiovasc Imaging 7:282-291. <https://doi.org/10.1161/circimaging.113.001047>

**6) Recruitment Period (2009–2020) and Temporal Heterogeneity
The recruitment period spans more than a decade. Between 2009 and 2020, substantial changes occurred in preventive cardiology and imaging: expansion of statin use, evolving lipid targets, increased BP awareness, and improvements in CCTA acquisition and reconstruction. Such temporal shifts may influence both the distribution of exposure and the detection and classification of CAD. It is unclear whether the year of examination was considered in the modelling and whether the associations were stable across time. Clarification of how the calendar year is handled in the analysis would strengthen confidence in the robustness of the findings. Consideration of temporal heterogeneity across the recruitment period, either analytically or within the Discussion, appears warranted.
This issue does not undermine the findings but warrants acknowledgement.
As with all observational cross-sectional analyses, residual confounding remains a plausible consideration, particularly given that cumulative BP exposure and certain lifestyle or socioeconomic factors may not be fully captured in the current dataset.**

**Author’s response:**

Thank you for this important comment. We acknowledge that the recruitment period of this study spanned more than a decade (2009-2020), during which changes in preventive cardiology practices and CCTA technology may have occurred. Because calendar year was not included as a covariate in the primary models, temporal changes in clinical practice or imaging technology during the study period cannot be completely excluded. We have therefore added a statement in the **Discussion** acknowledging the potential source of temporal heterogeneity.

**Discussion, page 13, 14**

In addition, the recruitment period of this study spanned more than a decade (2009-2020), during which changes in preventive cardiology practices and CCTA technology may have influenced the detection and classification of coronary atherosclerosis.

**7) Transparency of Study Population
Although the flow diagram presents exclusion criteria, the textual Methods section does not comprehensively describe all inclusion and exclusion criteria. For reproducibility and clarity, the Study Population subsection should explicitly list all inclusion and exclusion criteria and provide a rationale for key exclusions, especially those related to antihypertensive therapy. Transparent reporting is essential for interpretability.**

**Author’s response:**

We appreciate the Reviewer’s valuable comment regarding the transparency of the study population.

We agree that explicit reporting of all inclusion and exclusion criteria is essential for reproducibility and interpretability. In response to the Reviewer’s comment, we have revised the Methods section to clearly list all inclusion and exclusion criteria and to clarify the handling of antihypertensive medication use. Specifically, individuals receiving antihypertensive medication were excluded to avoid.

**Methods, 2.1. Study population, Page 6**

A total of 10,581 individuals aged ≥20 years who voluntarily underwent CCTA as part of a general health examination at the Health Promotion Center of Ulsan University Hospital (Ulsan, South Korea) between January 2009 and March 2020 were retrospectively included in the study. Inclusion criteria were (1) age ≥20 years and (2) asymptomatic status at the time of CCTA. Exclusion criteria were (1) insufficient medical records (n = 766), (2) history of angina or myocardial infarction and/or percutaneous coronary intervention (n = 340), (3) abnormal 12-lead electrocardiography (ECG) results (n = 90), (4) renal insufficiency (serum creatinine > 1.5mg/dL) (n = 47), (5) previous history of stroke (n = 42), (6) structural heart disease (n = 41), (7) history of open heart surgery (n = 18), (8) history of radiofrequency catheter ablation (n = 3), (9) history of patent foramen ovale device closure (n = 1), (10) poor image quality (n = 1), and (11) on anti-hypertensive medication (n = 1,900). After applying these criteria, 7,332 participants were included in the final analysis. The participant enrollment process is shown in **Figure 1**. This study was approved by the Institutional Review Board of Ulsan University Hospital (Ulsan, Korea), and the requirement for informed consent was waived due to its retrospective and cross-sectional design (IRB No. UUH 2020-12-033).

**Fig 1. Study flowchart**

**8) ASCVD Risk Score
The manuscript reports the calculation of 10-year ASCVD risk using pooled cohort equations. Additional clarification regarding the rationale for applying pooled cohort equations in this population would be appropriate. The role of ASCVD risk in the analysis, along with potential calibration limitations, merits a brief discussion.**

**Author’s response:**

We thank the Reviewer for this insightful comment regarding the use of the pooled cohort equations. In the present study, the 10-year ASCVD risk score was calculated solely to characterize the baseline cardiovascular risk profile of the study population and was presented as a descriptive variable in Table 1. Several individual components of the ASCVD risk score (such as age, sex, diabetes, and lipid levels) were already included as covariates in the multivariable models. Since incorporating the composite ASCVD risk score into the regression analysis may introduce potential collinearity, it was not used as a primary exposure, stratification variable, or covariate in the multivariable regression analyses.

Therefore, the ASCVD risk estimation did not directly influence the main analytic framework or the primary findings of this study. Although pooled cohort equations were originally developed in Western populations and may have calibration limitations in Asian cohorts, ASCVD risk was included for descriptive purposes only and was not central to the primary analysis. We hope that the reviewer will generously understand this.

**9) Depth of CCTA Phenotyping
The analysis relies on stenosis-based classification and CACS categories. Contemporary CCTA platforms allow more detailed plaque characterization. If plaque burden or composition data were unavailable due to retrospective constraints, this should be explicitly stated. Clarification regarding the availability of plaque burden or compositional metrics would be helpful. If such data were unavailable, this limitation should be explicitly acknowledged, particularly given that BP may correlate more closely with plaque burden than with binary stenosis thresholds.**

**Author’s response:**

We appreciate the Reviewer’s insightful comment regarding the depth of CCTA phenotyping. We agree that contemporary CCTA platforms allow more detailed plaque characterization, including quantitative assessment of plaque burden, plaque volume, and compositional features such as low-attenuation plaque and positive remodeling. These parameters may indeed provide additional mechanistic insights beyond binary stenosis classification or CACS categories.

However, due to the retrospective nature of our study and institutional data storage limitations, quantitative plaque burden and detailed plaque characterization were not systematically available for the entire cohort. At our institution, raw CCTA imaging data are retained only for a limited period. Therefore, comprehensive re-analysis of plaque burden and high-risk plaque features was not feasible for the entire cohort. Accordingly, our analysis was based on stenosis-based classification and plaque findings as documented in structured clinical reports. We acknowledge that the absence of quantitative plaque burden and detailed high-risk plaque characteristics represents an important limitation of our study.

In response to the Reviewer’s comment, we have revised the **Limitation section** to explicitly clarify this point.

**Discussion, Page 13**

Fifth, our study did not include detailed high-risk plaque characteristics, such as low-attenuation plaque or positive remodeling. Due to the retrospective design and limited availability of raw CCTA imaging data, comprehensive plaque phenotyping was not feasible. Future prospective studies with detailed quantitative and qualitative plaque analysis are warranted.

**10) Imaging-Based Vascular Phenotyping: Conceptual Refinement
This study has the potential to make a meaningful contribution to the concept of imaging-based vascular phenotyping. By focusing on untreated individuals and single-visit BP phenotype within a screening population, the study provides a snapshot of how current hemodynamic load relates to structural coronary remodelling. The Discussion would benefit from emphasising imaging-defined vascular phenotype, avoiding overextension into therapeutic inference, and framing the findings as characterisation of subclinical structural adaptation across BP strata. Such reframing would elevate the manuscript intellectually while remaining faithful to the data.**

**Author’s response:**

Thank you for this insightful suggestion.

We also appreciate the reviewer’s comment regarding the need to avoid overextension into therapeutic inference. Considering the cross-sectional design of the present study, we have revised several sentences in the **Abstract,** **Discussion** and **Conclusion sections** to avoid implying therapeutic effects. In addition, the interpretation of the findings has been framed more clearly as associations between blood pressure categories and imaging-defined subclinical coronary atherosclerosis.

**Abstract, Conclusions, page 3**

In this large cross-sectional study, the degree of hypertension was associated with an increased risk and severity of subclinical coronary atherosclerosis. These findings highlight the potential importance of early recognition of blood pressure elevation and cardiovascular risk stratification in asymptomatic individuals.

**Discussion, page 12**

Furthermore, long-term follow-up studies have shown that patients with non-obstructive CAD detected by CCTA are associated with an approximately two- to three-fold higher risk of all-cause mortality, nonfatal myocardial infarction, and subsequent revascularization [6, 33-36]. Our findings suggest that early hypertension may already be associated with subclinical coronary atherosclerosis and that a personalized approach is needed to reduce future coronary events in such early hypertensive individuals.

**Discussion, page 12**

This suggests that subclinical atherosclerosis may already be present even in patients traditionally classified as lower risk~~, underscoring potential gaps in existing recommendations and highlighting the need for more tailored preventive strategies in this population~~.

**Discussion, page 13**

Furthermore, in a large 10-year follow-up study, CCTA assessed CAD severity (normal, nonobstructive, or obstructive) and provided robust prognostic power for predicting cardiac death and nonfatal MI, clearly delineating diverging long-term risk trajectories and substantially improving risk reclassification [37]. Taken together, our findings suggest that increasing hypertension severity is associated with a greater burden of coronary atherosclerosis, highlighting the importance of early recognition of elevated blood pressure.

**Conclusion, page 14**

In conclusion, the degree of hypertension was significantly associated with the presence and severity of subclinical coronary atherosclerosis in asymptomatic individuals. These findings suggest that even early hypertension may be associated with subclinical coronary atherosclerosis, highlighting the importance of early recognition of blood pressure elevation and appropriate cardiovascular risk stratification.

**Final Recommendation
The study is methodologically sound and suitable for consideration in PLOS ONE. The requested revisions primarily concern clarification, proportional interpretation, and conceptual refinement. With appropriate revision, the manuscript would represent a methodologically sound and proportionately framed contribution within the scope of PLOS ONE.**

**Authors’ response:**

Thank you for your encouraging comments.

**Reviewer #4: Synopsys
This study evaluated the relationship between hypertension grade and subclinical coronary atherosclerosis in 7,332 asymptomatic individuals without known cardiovascular disease (mean age 52.8 ± 7.8 years; 63.8% men) who underwent CCTA. Participants were categorized as normal blood pressure (n=2,500), elevated (n=969), stage 1 hypertension (n=2,841), and stage 2 hypertension (n=1,022) according to ACC/AHA criteria. Coronary arteries were classified as normal, non-obstructive CAD (<50% stenosis), or obstructive CAD (≥50% stenosis). After adjustment for cardiovascular risk factors, stage 1 hypertension showed an association with non-obstructive CAD (aOR 1.335; 95% CI 1.156–1.541). Stage 2 hypertension showed associations with non-obstructive CAD (aOR 1.483; 95% CI 1.234–1.784) and obstructive CAD (aOR 1.696; 95% CI 1.194–2.409). Increasing hypertension category corresponded to higher prevalence of CAD.**

**General comment
1. The manuscript shows substantial overlap with the authors’ previous publication “Differential Impact of Degree of Hypertension on Subclinical Coronary Atherosclerosis in Asymptomatic Subjects With and Without Diabetes Mellitus” (DOI: 10.1016/j.amjcard.2023.07.036). The cohort appears to be essentially the same in size, demographics, and source population, and the core methodology (CCTA-based assessment of subclinical CAD and hypertension staging) is also largely unchanged. Although the present study does not stratify by diabetes status, the central message and direction of the associations are highly similar to those already reported. In its current form, the incremental value seems limited. To justify a separate publication, the authors should clearly state the degree of dataset overlap with prior work, explicitly delineate what is genuinely new in terms of hypothesis and analysis, and better articulate the additional clinical or scientific value provided by this study beyond their earlier report. Strengthening these aspects would help clarify the distinct contribution of the manuscript.**

**Author’s response:**

Thank you for this insightful comment. We acknowledge that the present study and our previous publication (PMID: 37517130) were derived from the same health screening CCTA registry. However, the objectives, analytic focus, and outcome definitions of the two studies differ substantially.

The previous study primarily aimed to determine whether diabetes mellitus modifies the relationship between hypertension and subclinical coronary atherosclerosis. Accordingly, the analysis was stratified by diabetes status, and the primary outcome was defined as the presence of coronary plaque detected on CCTA.

In contrast, the present study focuses on the relationship between graded blood pressure categories and the severity and burden of coronary artery disease detected by CCTA in asymptomatic individuals without prior cardiovascular disease. Rather than evaluating diabetes-related effect modification, this analysis examines imaging-based indicators of disease severity, including non-obstructive CAD, obstructive CAD, and coronary artery calcium burden across blood pressure categories.

Therefore, while both studies share the same registry, they address different clinical questions and analytic perspectives, providing complementary insights into the relationship between blood pressure and subclinical coronary atherosclerosis.

**Reviewer #5: Park et al evaluated the correlation of arterial hypertension (HTN) and coronary atherosclerosis (CAD) assessed by computed tomography angiography (CTA) in a large cohort of asymptomatic patients. The main finding of the study is that stage 1 HTN was independently associated with non-obstructive CAD and stage 2 HTN with both non-obstructive and obstructive CAD.
This work was designed as a retrospective analysis of a large prospective cohort. The text is generally well written, easy to read and completely understandable. Methods, results and discussion sections are well described, presented and commented on. In any case, some questions need to be addressed.**

**1. First of all, this study encompasses a large cohort of asymptomatic individuals undergoing CTA as part of “a general health examination”. As this was not a clinical trial and CTA is only indicated in selected high risk asymptomatic patients(1), a further explanation of its indication would be desirable.**

**Author’s response:**

We thank the reviewer for this important comment.

As a compulsory social insurance, South Korea has a National Health Insurance (NHI) system which covers the whole population living in the country. NHI has actively operated the business of promoting health checkups and health level in an effort to detect diseases early and enhance public health (**Figure 1**). These general health screenings have been performed to the employee insured with no age limit (annually) and the self-employed insured such as householders or dependents older than 40 year-old (biannually). These general checkups are free and covered by NIH. Anyone can receive general medical checkups at any hospital in the country. These general heath checkups include past and current medical history including smoking and alcohol status, exercise status, family medical history, physical examination, height, weight, waist circumference, systolic and diastolic blood pressure, blood tests (fasting blood glucose, fasting total cholesterol, high-density lipoprotein cholesterol, low-density lipoprotein cholesterol, triglycerides, hemoglobin, creatinine, aspartate transaminase [AST], alanine transferase,[ALT], gamma glutamyl transferase), urinalysis, and chest X-ray. All general heath checkups are reviewed and examined by doctors.

The present study included individuals who had these general medical checkups. Because Ulsan University Hospital is the only Tertiary Hospital in Ulsan Metropolitan City, this hospital holds a lot of health information of individuals who had voluntarily undergone a general health examination. In addition, if individuals undergoing general medical checkups paid additional costs, they could take additional blood or imaging tests such as coronary computed tomography angiography (CCTA), abdominal ultrasonography, brain MRI and so on.

In the present study, the study population consisted of 10,581 consecutive asymptomatic individuals who voluntarily underwent self-referred CCTA as part of a general health examination at the Health Promotion Center of Ulsan University Hospital. At the time of the examination, the potential risks associated with CCTA, including radiation exposure and contrast use, were explained to all participants.

Therefore, the primary reason for performing CCTA in this cohort was voluntary screening for the early detection of subclinical coronary artery disease within a health examination program, rather than symptom-driven or physician-directed diagnostic evaluation.

**Figure 1.** NHI Health promotion system in South Korea

**References**

1. National Health Insurance Service. https://www.nhis.or.kr/english/index.do

**2. Regarding CTA analysis some additional data should be included. Calcium score was reported and analyzed by categories. However, it is known that the values are highly dependent on age and sex; therefore, an additional analysis considering the percentile(2) would be interesting. Current SCCT guidelines(3) recommend a quantitative stenosis grading of 5 categories and the use of the CAD-RADS 2.0 classification system(4). The limited classification of obstructive (<50%) and non-obstructive (>50%) CAD precludes a evaluation of the importance of HTN in the CAD burden. Thus, a detailed coronary stenosis grading should be performed and analyzed.**

**Author’s response:**

We thank the Reviewer for this thoughtful comment regarding the depth of coronary stenosis characterization. We agree that contemporary CCTA reporting systems, including the SCCT-recommended 5-category stenosis grading and the CAD-RADS classification, allow a more refined assessment of disease severity and may provide additional insight into the gradient of coronary atherosclerotic burden.

However, in our study, stenosis severity was documented in structured clinical reports using a binary classification (<50% vs ≥50%), and detailed percentage-based stenosis grading was not systematically recorded for the entire cohort. Due to the retrospective design and limited availability of raw CCTA imaging data, reclassification into SCCT 5-grade stenosis categories or CAD-RADS 2.0 categories was not feasible for the entire cohort.

Importantly, the ≥50% threshold was selected because it represents a clinically established definition of obstructive coronary artery disease and has consistently demonstrated prognostic value in large CCTA registries and long-term outcome studies. Prior studies have shown that both non-obstructive and obstructive CAD defined by this threshold are associated with stepwise increases in adverse cardiovascular outcomes [1-3]. Therefore, although more detailed stenosis grading may offer incremental mechanistic insight, the dichotomized classification remains clinically meaningful and appropriate for risk stratification. In response to the Reviewer’s comment, we have revised the **Limitation section** to explicitly clarify this point.

**Discussion, Page 13**

Fifth, our study did not include detailed high-risk plaque characteristics, such as low-attenuation plaque or positive remodeling. Due to the retrospective design and limited availability of raw CCTA imaging data, comprehensive plaque phenotyping was not feasible. Future prospective studies with detailed quantitative and qualitative plaque analysis are warranted.

**References**

1.	Bittencourt MS, Hulten E, Ghoshhajra B, O'Leary D, Christman MP, Montana P, et al. Prognostic value of nonobstructive and obstructive coronary artery disease detected by coronary computed tomography angiography to identify cardiovascular events. Circ Cardiovasc Imaging. 2014;7(2):282-91. Epub 20140218. doi: 10.1161/circimaging.113.001047. PubMed PMID: 24550435.

2.	Lin FY, Shaw LJ, Dunning AM, Labounty TM, Choi JH, Weinsaft JW, et al. Mortality risk in symptomatic patients with nonobstructive coronary artery disease: a prospective 2-center study of 2,583 patients undergoing 64-detector row coronary computed tomographic angiography. J Am Coll Cardiol. 2011;58(5):510-9. doi: 10.1016/j.jacc.2010.11.078. PubMed PMID: 21777749.

3.	Cheruvu C, Precious B, Naoum C, Blanke P, Ahmadi A, Soon J, et al. Long term prognostic utility of coronary CT angiography in patients with no modifiable coronary artery disease risk factors: Results from the 5 year follow-up of the CONFIRM International Multicenter Registry. J Cardiovasc Comput Tomogr. 2016;10(1):22-7. Epub 20151215. doi: 10.1016/j.jcct.2015.12.005. PubMed PMID: 26719237; PubMed Central PMCID: PMCPMC4869323.

**3. Univariate as well as multivariate analysis reveal that not only HTN but almost all the rest of cardiovascular risk factors were associated with CAD in CTA. ASCVD risk score was ASCVD risk score was associated with the different categories of blood pressure, but it was included nor in the univariate neither in the multivariate analysis of CAD. Although the aim of the study was to determine the independent relevance of HTN, over the other risk factors, an specific analysis of ASCVD risk score should be performed.**

**Author’s response:**

Thank you for your valuable comment.

We agree that incorporating the ASCVD risk score may provide additional insight into the overall cardiovascular risk burden in relation to coronary artery disease. However, in our study, the ASCVD risk score was calculated and presented in the baseline characteristics to provide descriptive information on overall cardiovascular risk burden across blood pressure categories. Since several individual components of the ASCVD risk score (such as age, sex, diabetes, and lipid levels) were already included as covariates in the multivariable models, incorporating the composite ASCVD risk score into the regression analysis may introduce potential collinearity. For these reasons, the ASCVD risk score was not included in the univariable or multivariable regression analyses. We hope that the reviewer will generously understand this.

**In summary, this study reinforces the independent association of HTN with CAD evaluated by CTA. Nonetheless, aforementioned aspects related with patient population and methodology need a further explanation.**

**REFERENCES**
1. Narula J, Chandrashekhar Y, Ahmadi A, Abbara S, Berman DS, Blankstein R, et al. SCCT 2021 Expert Consensus Document on Coronary Computed Tomographic Angiography: A Report of the Society of Cardiovascular Computed Tomography. J Cardiovasc Comput Tomogr. 2021;15(3):192-217.
2. McClelland RL, Chung H, Detrano R, Post W, Kronmal RA. Distribution of coronary artery calcium by race, gender, and age: results from the Multi-Ethnic Study of Atherosclerosis (MESA). Circulation. 2006;113(1):30-7.
3. Raff GL, Abidov A, Achenbach S, Berman DS, Boxt LM, Budoff MJ, et al. SCCT guidelines for the interpretation and reporting of coronary computed tomographic angiography. J Cardiovasc Comput Tomogr. 2009;3(2):122-36.
4. Cury RC, Leipsic J, Abbara S, Achenbach S, Berman D, Bittencourt M, et al. CAD-RADS 2.0 - 2022 Coronary Artery Disease-Reporting and Data System: An Expert Consensus Document of the Society of Cardiovascular Computed Tomography (SCCT), the American College of Cardiology (ACC), the American College of Radiology (ACR), and the North America Society of Cardiovascular Imaging (NASCI). J Cardiovasc Comput Tomogr. 2022;16(6):536-57.

**Reviewer #6: This manuscript presents a large cross-sectional analysis of asymptomatic individuals undergoing CCTA and evaluates the association between hypertension severity and subclinical coronary atherosclerosis. The research question is clearly defined and clinically relevant. The sample size is substantial and imaging assessment appears rigorous. The findings are generally supported by the presented data.

However, several methodological aspects require clarification to ensure transparency and scientific rigor:

1. Antihypertensive medication status: The flow diagram suggests exclusion of individuals receiving antihypertensive therapy, yet the Methods section does not clearly describe how treated hypertension was handled. Clarification is needed regarding inclusion/exclusion criteria and classification of treated individuals, as this may influence interpretation.**

**Author’s response:**

We appreciate the Reviewer’s valuable comment.

We agree that explicit reporting of inclusion and exclusion criteria is essential for reproducibility and interpretability. In response to the Reviewer’s comment, we have revised the **Methods section** to clearly list all inclusion and exclusion criteria. Specifically, individuals receiving antihypertensive medication were excluded from the study population. The corresponding exclusion criteria have also been clarified in **Figure 1**.

**Methods, 2.1. Study population, Page 6**

A total of 10,581 individuals aged ≥20 years who voluntarily underwent CCTA as part of a general health examination at the Health Promotion Center of Ulsan University Hospital (Ulsan, South Korea) between January 2009 and March 2020 were retrospectively included in the study. Inclusion criteria were (1) age ≥20 years and (2) asymptomatic status at the time of CCTA. Exclusion criteria were (1) insufficient medical records (n = 766), (2) history of angina or myocardial infarction and/or percutaneous coronary intervention (n = 340), (3) abnormal 12-lead electrocardiography (ECG) results (n = 90), (4) renal insufficiency (serum creatinine > 1.5mg/dL) (n = 47), (5) previous history of stroke (n = 42), (6) structural heart disease (n = 41), (7) history of open heart surgery (n = 18), (8) history of radiofrequency catheter ablation (n = 3), (9) history of patent foramen ovale device closure (n = 1), (10) poor image quality (n = 1), and (11) on anti-hypertensive medication (n = 1,900). After applying these criteria, 7,332 participants were included in the final analysis. The participant enrollment process is shown in **Figure 1**. This study was approved by the Institutional Review Board of Ulsan University Hospital (Ulsan, Korea), and the requirement for informed consent was waived due to its retrospective and cross-sectional design (IRB No. UUH 2020-12-033).

**Fig 1. Study flowchart**

**2. Blood pressure classification: BP was measured during a single health screening visit. Although repeated measurements during the visit were averaged appropriately, discussion of potential misclassification (e.g., white coat effect or previously diagnosed hypertension) would strengthen interpretability.**

**Author’s response:**

We thank the Reviewer for this insightful comment regarding blood pressure classification. Blood pressure was measured during a single health screening visit. To enhance measurement reliability, systolic and diastolic blood pressures were recorded three times, and the average of the second and third measurements was used for analysis. We acknowledge, however, that single-visit office blood pressure measurements may not fully reflect an individual’s usual blood pressure. In addition, out-of-office measurements such as home blood pressure or ambulatory blood pressure monitoring were not available in this cohort. Therefore, the possibility of white coat hypertension or masked hypertension cannot be excluded. In response to the Reviewer’s suggestion, we have revised the **Limitation** section to explicitly acknowledge this issue.

**Discussion, Page 13**

Sixth, blood pressure was assessed during a single health screening visit. Although systolic and diastolic blood pressures were measured three times and the average of the second and third readings was used to improve reliability, single-visit measurements may not fully reflect usual blood pressure levels. Furthermore, out-of-office measurements such as home blood pressure or ambulatory blood pressure monitoring were not available. Therefore, the possibility of white coat hypertension or masked hypertension cannot be excluded.

**3. Multivariable model specification: While the included covariates are clinically appropriate, additional transparency regarding model selection strategy, assessment of multicollinearity, and rationale for not including ASCVD risk score would improve methodological clarity.**

**Author’s response:**

We thank the Reviewer for this valuable comment regarding multivariable model specification. Covariates were selected a priori based on clinical relevance and established cardiovascular risk factors. Specifically, age, sex, obesity, diabetes mellitus, dyslipidemia, current smoking, and family history of CAD were included as potential confounders based on their well-established associations with coronary artery disease [1].

We assessed multicollinearity among covariates using variance inflation factors (VIFs), and no evidence of problematic multicollinearity was observed (all VIFs < 2.0; **Table**).

We did not include the ASCVD risk score in the regression model because it combines several overlapping cardiovascular risk factors, including age, sex, blood pressure, lipid parameters, diabetes, and smoking status. Therefore, we adjusted for individual clinically relevant risk factors to maintain consistency and interpretability across the entire cohort.

We believe these clarifications improve the transparency of the model specification. We hope that the reviewer will generously understand this.

**Table. Variance inflation factors (VIFs) for covariates included in the multivariable regression model.**

| **Covariate** | **Collinearity Statistics** | |
| --- | --- | --- |
|  | **Tolerance** | **VIF** |
| Age | 0.958 | 1.043 |
| Sex | 0.848 | 1.179 |
| Obesity (BMI ≥ 25 kg/m^2)_^ | 0.968 | 1.033 |
| Diabetes mellitus | 0.975 | 1.026 |
| Dyslipidemia | 0.997 | 1.003 |
| Current smoking | 0.853 | 1.173 |
| Family history of CAD | 0.996 | 1.004 |

**Reference**

1. Goff DC Jr, Lloyd-Jones DM, Bennett G, et al. 2013 ACC/AHA guideline on the assessment of cardiovascular risk: a report of the American College of Cardiology/American Heart Association Task Force on Practice Guidelines. J Am Coll Cardiol. 2014 Jul 1;63(25 Pt B):2935-2959.

**4. Cross-sectional design: The study demonstrates association rather than causation. The Discussion would benefit from explicitly reinforcing this distinction.**

**Author’s response:**

We thank the Reviewer for this important comment. We fully agree that, given the cross-sectional design, the findings should be interpreted as associations rather than causal relationships. As stated in the **Limitation section** of the discussion, we have explicitly acknowledged that causality cannot be inferred and that residual confounding cannot be fully excluded despite multivariable adjustment. This distinction between association and causation has been clearly addressed in the revised manuscript.

**Discussion, Page 13**

This study has several limitations. First, this was a single-center study conducted on Korean individuals, which limits the generalizability of the findings to other ethnic groups. Second, because the study population consisted of individuals who voluntarily underwent health screening with CCTA, a degree of selection bias may be inevitable, and the results may not be fully generalizable to the general population. **Third, given the cross-sectional design, causal relationships cannot be inferred, and residual confounding cannot be fully excluded, despite multivariable adjustment**.

**5. Elevated BP category: The loss of statistical significance after adjustment warrants brief discussion to aid interpretation.**

**Author’s response:**

We thank the Reviewer for this thoughtful comment. As suggested, we have added a brief explanation in the **Discussion** section regarding the attenuation of the association in the elevated BP category after multivariable adjustment.

**Discussion, Page 10, 11**

In this large cohort of asymptomatic individuals without a prior history of cardiovascular disease, hypertension severity was significantly associated with the prevalence of subclinical coronary atherosclerosis. Both stage 1 and stage 2 hypertension were independently associated with non-obstructive CAD, while stage 2 hypertension was additionally associated with obstructive CAD. These findings are consistent with prior epidemiological studies demonstrating a graded association between blood pressure and coronary atherosclerosis and extend this evidence through imaging-based assessment in an asymptomatic screening population [11, 25]. In contrast, the association observed in the elevated BP category was attenuated after multivariable adjustment, suggesting that the independent effect of BP within this range may be limited after accounting for other cardiovascular risk factors. However, taken together, these findings highlight the potential importance of early recognition of blood pressure elevation in asymptomatic populations.

Overall, the manuscript addresses a relevant research question and is potentially suitable for publication after clarification of the above points.

6. PLOS authors have the option to publish the peer review history of their article ([what does this mean?](https://track.editorialmanager.com/CL0/https:%2F%2Fjournals.plos.org%2Fplosone%2Fs%2Feditorial-and-peer-review-process%23loc-peer-review-history/1/010f019c93c0c3e1-249079e6-a3ac-47a5-89e8-064ceca5b0fb-000000/eAUurV_4DULZ8qHmw_Y5r3sHxSu5HiswgSYs776M3T4=252)). If published, this will include your full peer review and any attached files.


If you choose “no”, your identity will remain anonymous but your review may still be made public.


**Do you want your identity to be public for this peer review?** For information about this choice, including consent withdrawal, please see our [Privacy Policy](https://track.editorialmanager.com/CL0/https:%2F%2Fwww.plos.org%2Fprivacy-policy/1/010f019c93c0c3e1-249079e6-a3ac-47a5-89e8-064ceca5b0fb-000000/gwhoF8nbLu7onVk-SXy7XDw2UND2sjuHVAtqtFwlvKA=252).

Reviewer #1: No

Reviewer #2: No

Reviewer #3: **Yes:** Rafal Dankowski

Reviewer #4: No

Reviewer #5: No

Reviewer #6: No

[NOTE: If reviewer comments were submitted as an attachment file, they will be attached to this email and accessible via the submission site. Please log into your account, locate the manuscript record, and check for the action link "View Attachments". If this link does not appear, there are no attachment files.]

To ensure your figures meet our technical requirements, please review our figure guidelines: <https://journals.plos.org/plosone/s/figures>

You may also use PLOS’s free figure tool, NAAS, to help you prepare publication quality figures: <https://journals.plos.org/plosone/s/figures#loc-tools-for-figure-preparation.>

NAAS will assess whether your figures meet our technical requirements by comparing each figure against our figure specifications.
