# Supplementary material for: Degree of hypertension and subclinical coronary atherosclerosis in asymptomatic individuals without cardiovascular disease
Source: PLoS One. 2026 Jul 10;21(7):e0353359. doi: 10.1371/journal.pone.0353359 (PMC13354002; doi:10.1371/journal.pone.0353359)
Supplement: Attachment — Submitted filename: Response_to_Reviewers_auresp_2.docx [file pone.0353359.s003.docx]

**Journal Requirements:**

**1. If the reviewer comments include a recommendation to cite specific previously published works, please review and evaluate these publications to determine whether they are relevant and should be cited. There is no requirement to cite these works unless the editor has indicated otherwise.**

**Author’s response:**

Thank you for this helpful guidance. We have carefully reviewed the suggested references and evaluated their relevance. Only appropriate and relevant studies have been included in the revised manuscript.

**2. Please review your reference list to ensure that it is complete and correct. If you have cited papers that have been retracted, please include the rationale for doing so in the manuscript text, or remove these references and replace them with relevant current references. Any changes to the reference list should be mentioned in the rebuttal letter that accompanies your revised manuscript. If you need to cite a retracted article, indicate the article’s retracted status in the References list and also include a citation and full reference for the retraction notice.**

**Author’s response:**

**Thank you for this important comment.**

We have thoroughly reviewed the reference list to ensure accuracy, completeness, and relevance. No retracted articles were identified.

Additional Editor Comments:

Please, address the comments raised by Reviewer 2

[Note: HTML markup is below. Please do not edit.]

**Reviewers' comments:**

Reviewer's Responses to Questions

Comments to the Author

1. If the authors have adequately addressed your comments raised in a previous round of review and you feel that this manuscript is now acceptable for publication, you may indicate that here to bypass the “Comments to the Author” section, enter your conflict of interest statement in the “Confidential to Editor” section, and submit your "Accept" recommendation.

Reviewer #2: (No Response)

Reviewer #3: All comments have been addressed

Reviewer #5: All comments have been addressed

2. Is the manuscript technically sound, and do the data support the conclusions?

The manuscript must describe a technically sound piece of scientific research with data that supports the conclusions. Experiments must have been conducted rigorously, with appropriate controls, replication, and sample sizes. The conclusions must be drawn appropriately based on the data presented.

Reviewer #2: Partly

Reviewer #3: Yes

Reviewer #5: Yes

3. Has the statistical analysis been performed appropriately and rigorously?

Reviewer #2: No

Reviewer #3: Yes

Reviewer #5: Yes

4. Have the authors made all data underlying the findings in their manuscript fully available?

The [PLOS Data policy](http://track.editorialmanager.com/CL0/http:%2F%2Fwww.plosone.org%2Fstatic%2Fpolicies.action%23sharing/1/010f019ddcc68816-bd9f8d7a-97eb-4a2e-955c-1d7ac5776327-000000/hoNdfp560inHrGv3ieuunVvTP3sbLVz8aYvA9ZzS44A=258) requires authors to make all data underlying the findings described in their manuscript fully available without restriction, with rare exception (please refer to the Data Availability Statement in the manuscript PDF file). The data should be provided as part of the manuscript or its supporting information, or deposited to a public repository. For example, in addition to summary statistics, the data points behind means, medians and variance measures should be available. If there are restrictions on publicly sharing data—e.g. participant privacy or use of data from a third party—those must be specified.

Reviewer #2: Yes

Reviewer #3: No

Reviewer #5: Yes

5. Is the manuscript presented in an intelligible fashion and written in standard English?

PLOS ONE does not copyedit accepted manuscripts, so the language in submitted articles must be clear, correct, and unambiguous. Any typographical or grammatical errors should be corrected at revision, so please note any specific errors here.

Reviewer #2: Yes

Reviewer #3: Yes

Reviewer #5: Yes

6. Review Comments to the Author

Please use the space provided to explain your answers to the questions above. You may also include additional comments for the author, including concerns about dual publication, research ethics, or publication ethics. (Please upload your review as an attachment if it exceeds 20,000 characters)

**Reviewer #2: I thank the authors for the revisions made in response to my previous comments. While the manuscript has improved in several aspects, there remain some important issues that need to be addressed.**

**1) In response to my comment regarding the previous studies by Kang and Im, the authors cited these studies and provided a detailed explanation of the differences between the present study and previous ones. However, these differences were described only as “Previous studies in asymptomatic populations have similarly reported higher coronary calcium burden with increasing blood pressure severity, although differences exist in BP classification systems and analytic approaches” in the text. I suggest that the authors incorporate a "shorter version" of their response (1) into the Discussion section, which will emphasize the new findings of this study and strengthen the conclusions.**

**Author’s response:**

Thank you for these valuable comments.

In response to the reviewer’s suggestion, we have incorporated a concise version of our previous explanation into the Discussion section to better highlight the differences between our study and prior studies. We believe that this modification improves the clarity of the manuscript and strengthens the interpretation of our findings.

**Discussion, page 11**

Previous studies in asymptomatic populations have similarly reported higher coronary calcium burden with increasing blood pressure severity, although differences exist in BP classification systems and analytic approaches [27, 28]. Specifically, earlier studies such as those by Im et al. applied the Seventh Report of the Joint National Committee (JNC 7) classification, whereas our study used contemporary ACC/AHA guidelines. Moreover, while prior studies mainly evaluated the presence of coronary artery calcium, we assessed coronary artery calcium burden in greater detail and further evaluated luminal stenosis severity using CCTA.

**2) In the response to my comment 2, the authors gave the detailed reasons and the background of performing coronary CCTA in 10,581 asymptomatic subjects. I am satisfied with this explanation, but the readers of this article may have the same question as I had. The authors should add a brief explanation of the background of performing CCTA as a part of general medical checkups, which will increase the readability of the article. If CCTA is performed only in subjects who pay additional cost, it may introduce selection bias into the study population.**

**Author’s response:**

Thank you for this valuable comment. In response to the reviewer’s comment, we have now added a brief explanation of this background in the Methods section to improve clarity for readers. We also acknowledge that such self-selection may introduce potential selection bias, which has been addressed in the Discussion section.

**Methods, Study population, page 6**

South Korea has a National Health Insurance system. The National Health Insurance in South Korea has actively operated the business of promoting health checkups and health levels in an effort to detect diseases early and enhance public health. These general health screenings have been performed on the employee insured with no age limit (annually) and the self-employed insured, such as householders or dependents aged >40 years (biannually). These general checkups are free and covered by the National Health Insurance. In addition, if individuals undergoing general medical checkups paid additional costs, they could take additional tests, such as CCTA. In this study, a total of 10,581 individuals aged ≥20 years who voluntarily underwent CCTA as part of a general health examination at the Health Promotion Center of Ulsan University Hospital (Ulsan, South Korea) between January 2009 and March 2020 were retrospectively included in the study.

**Discussion, page 13**

First, this was a single-center study conducted on Korean individuals, which limits the generalizability of the findings to other ethnic groups. Second, because the study population consisted of individuals who voluntarily underwent health screening with CCTA, a degree of selection bias may be inevitable, and the results may not be fully generalizable to the general population.

**3) As previously pointed out, there are differences in many clinical characteristics, including age and sex, between the 4 groups (Table 1), and their influence must be analyzed carefully. The authors performed interaction analyses to evaluate whether the association between hypertension stage and CAD outcomes differed according to sex, age group, and obesity. They found a significant interaction between hypertension stage and age, but no significant interactions for sex or obesity in relation to non-obstructive CAD. However, these results may not be consistent with the well-known sex difference in CAC development, with women developing calcification approximately 10–20 years later than men at similar absolute CAC scores. Actually, this sex difference profoundly affects CAC interpretation, particularly in younger subjects where age-sex-race percentiles become essential for proper risk assessment.
The authors should provide a more detailed analysis of the influence of age and sex (or their interaction) on the development of CAC.**

**Author’s response:**

Thank you for your valuable comments. We fully agree that age and sex are important determinants of coronary artery calcium, and that CAC should be interpreted carefully in the context of age-and sex-specific differences. In the present study, age and sex were included as covariates in the multivariable models to account for their potential confounding effects on the association between hypertension stage and subclinical coronary atherosclerosis. In addition, CAC burden according to hypertension stage was presented in Table 2, showing a graded increase in CAC categories with higher hypertension stages.

While we acknowledge the importance of age- and sex-specific interpretation of CAC, the present analysis was primary designed to evaluate the association between hypertension stage and subclinical coronary atherosclerosis. Therefore, detailed analyses focusing on the modifying effects of age and sex on CAC were beyond the scope of the present study.

Furthermore, the absence of a significant interaction with sex in our CAD-based analyses should not be interpreted as evidence that there are no sex-related differences in coronary atherosclerosis. Rather, it indicates that the relative association between hypertension stage and CAD outcomes was not significantly different between men and women.

**4) In the response to my comment on the CACS category, the authors provided the results of analysis based on the 4-group classification (CACS 0, 1-99, 100-299 and ≥300), which is a widely used classification in studies and in clinical practice, which was consistent with the results based on the 5-group classification. On the other hand, the authors provided the results of an analysis based on a 4-group classification (CACS 0, 1–99, 100–299, and ≥300), which is widely used in studies and clinical practice, and which was consistent with the results based on the 5-group classification. What is the rationale for dividing those with CACS 1–100 into 2 groups?**

**Author’s response:**

Thank you for this important comment. We agree that the 4-group classification (0, 1-99, 100-299, ≥300) is widely used in both clinical practice and prior studies. In the present study, we further subdivided the 1-100 range into 1-10 and 11-100 categories to allow a more detailed assessment of low coronary artery calcium burden. As shown in prior studies, CACS 1-10 is generally considered minimal plaque, whereas 11-100 represents at least mild atherosclerotic plaque, reflecting differences in early disease burden [1, 2].

As our study population consisted of asymptomatic individuals undergoing health screening, this stratification was intended to better characterize the distribution of CACS. Importantly, the overall trends and associations remained consistent regardless of whether the 4-group or 5-group classification was applied.

**[Reference]**

1. Agatston AS, Janowitz WR, Hildner FJ, Zusmer NR, Viamonte M, Jr., Detrano R. Quantification of coronary artery calcium using ultrafast computed tomography. J Am Coll Cardiol. 1990;15(4):827-32. doi: 10.1016/0735-1097(90)90282-t. PubMed PMID: 2407762.

2. Rumberger JA, Brundage BH, Rader DJ, Kondos G. Electron beam computed tomographic coronary calcium scanning: a review and guidelines for use in asymptomatic persons. Mayo Clin Proc. 1999;74(3):243-52. doi: 10.4065/74.3.243. PubMed PMID: 10089993.

**Reviewer #3: Thank you for the revised version of the manuscript. The authors have made a clear effort to address the methodological and conceptual points raised in the initial review, and the manuscript has improved as a result.
In particular, clarifying blood pressure classification based on single-visit measurements, along with an expanded discussion of its limitations, meaningfully strengthens the interpretability of the findings. The manuscript is now more transparent regarding the study design, statistical approach, and the scope of available imaging data, which improves overall clarity and reproducibility.
The revisions related to statistical modelling, study population, and imaging limitations are appropriate and sufficient for the scope of this analysis. The authors have also made a clear effort to align the interpretation of the results with the observational nature of the data, thereby improving the internal consistency of the manuscript.
One point that may still be worth keeping in mind is that some parts of the discussion are still somewhat more interpretative than the data allow. Given the cross-sectional design, maintaining consistently cautious language throughout the discussion and conclusions would further strengthen the manuscript and ensure that the interpretation remains fully aligned with the study design.
Overall, the manuscript now represents a methodologically sound and clearly presented analysis. I have no major concerns at this stage.**

**Author’s response:**

Thank you for your valuable comments. We sincerely appreciate your positive feedback on the revised manuscript. We agree with your suggestion regarding the need for consistently cautious interpretation, particularly given the cross-sectional nature of the study. Accordingly, we have carefully revised the Discussion and Conclusions to ensure that the interpretation remains appropriately aligned with the study design. We are grateful for your valuable feedback, which has further strengthened the clarity and rigor of our manuscript.

**Discussion, page 12**

Our findings suggest that early hypertension may already be associated with subclinical coronary atherosclerosis and may have implications for personalized risk assessment and prevention strategies in these individuals.

**Discussion, page 13**

Importantly, stage 2 hypertension was independently associated with both non-obstructive and obstructive CAD, suggesting that more advanced blood pressure elevation might be associated with not only early atherosclerotic changes but also clinically significant coronary disease.

**Conclusion, page 14**

These findings suggest that even early hypertension may be associated with subclinical coronary atherosclerosis, highlighting the potential importance of early recognition of blood pressure elevation and appropriate cardiovascular risk stratification.

**Reviewer #5: The authors have included the answers to the previously made questions along the article. A more detailed plaque analysis would be desirable, but unfortunately the authors argue that those data are no longer available. Moreover, there is still concern regarding the potential selection bias secondary to the autorreferal of the evaluated individuals to computed tomography angiography (CTA). In any case, even with those limitations the paper has been able to demonstrate that even at early stages, arterial hypertension is associated with coronary atherosclerosis. Therefore, I consider that this work is clinically relevant.**

**Author’s response:**

Thank you for your valuable comments. We sincerely appreciate your positive evaluation of our work and your recognition of its clinical relevance.

We acknowledge the limitations regarding the lack of detailed plaque analysis and the potential for selection bias related to the self-referral of participants for coronary computed tomography angiography, and these points have been further clarified in the revised manuscript.

7. PLOS authors have the option to publish the peer review history of their article ([what does this mean?](https://track.editorialmanager.com/CL0/https:%2F%2Fjournals.plos.org%2Fplosone%2Fs%2Feditorial-and-peer-review-process%23loc-peer-review-history/1/010f019ddcc68816-bd9f8d7a-97eb-4a2e-955c-1d7ac5776327-000000/el5BMSpJx3uypkTJzI5eO7A7PlDWee5CjX9Qibz7VuE=258)). If published, this will include your full peer review and any attached files.


If you choose “no”, your identity will remain anonymous but your review may still be made public.


Do you want your identity to be public for this peer review? For information about this choice, including consent withdrawal, please see our [Privacy Policy](https://track.editorialmanager.com/CL0/https:%2F%2Fwww.plos.org%2Fprivacy-policy/1/010f019ddcc68816-bd9f8d7a-97eb-4a2e-955c-1d7ac5776327-000000/AZ-z-jhYqF5b5NGSTfsA0haw0qqifs0uDrh3iyoZqbk=258).

Reviewer #2: No

Reviewer #3: No

Reviewer #5: No

[NOTE: If reviewer comments were submitted as an attachment file, they will be attached to this email and accessible via the submission site. Please log into your account, locate the manuscript record, and check for the action link "View Attachments". If this link does not appear, there are no attachment files.]

To ensure your figures meet our technical requirements, please review our figure guidelines: <https://journals.plos.org/plosone/s/figures>

You may also use PLOS’s free figure tool, NAAS, to help you prepare publication quality figures: <https://journals.plos.org/plosone/s/figures#loc-tools-for-figure-preparation.>

NAAS will assess whether your figures meet our technical requirements by comparing each figure against our figure specifications.
