# Supplementary material for: Degree of hypertension and subclinical coronary atherosclerosis in asymptomatic individuals without cardiovascular disease
Source: PLoS One. 2026 Jul 10;21(7):e0353359. doi: 10.1371/journal.pone.0353359 (PMC13354002; doi:10.1371/journal.pone.0353359)
Supplement: Attachment — Submitted filename: Response_to_Reviewers_auresp_3.docx [file pone.0353359.s004.docx]

**Journal Requirements:**

**Journal Requirements:**

**1. If the reviewer comments include a recommendation to cite specific previously published works, please review and evaluate these publications to determine whether they are relevant and should be cited. There is no requirement to cite these works unless the editor has indicated otherwise.**

**Author’s response:**

Thank you for this helpful guidance. We have carefully reviewed the suggested references and evaluated their relevance. Only appropriate and relevant studies have been included in the revised manuscript.

**2. Please review your reference list to ensure that it is complete and correct. If you have cited papers that have been retracted, please include the rationale for doing so in the manuscript text, or remove these references and replace them with relevant current references. Any changes to the reference list should be mentioned in the rebuttal letter that accompanies your revised manuscript. If you need to cite a retracted article, indicate the article’s retracted status in the References list and also include a citation and full reference for the retraction notice.**

**Author’s response:**

**Thank you for this important comment.**

We have thoroughly reviewed the reference list to ensure accuracy, completeness, and relevance. No retracted articles were identified.

[Note: HTML markup is below. Please do not edit.]
 **Reviewers' comments:**

Reviewer's Responses to Questions

Comments to the Author

1. If the authors have adequately addressed your comments raised in a previous round of review and you feel that this manuscript is now acceptable for publication, you may indicate that here to bypass the “Comments to the Author” section, enter your conflict of interest statement in the “Confidential to Editor” section, and submit your "Accept" recommendation.

Reviewer #2: (No Response)

2. Is the manuscript technically sound, and do the data support the conclusions?

The manuscript must describe a technically sound piece of scientific research with data that supports the conclusions. Experiments must have been conducted rigorously, with appropriate controls, replication, and sample sizes. The conclusions must be drawn appropriately based on the data presented.

Reviewer #2: Yes

3. Has the statistical analysis been performed appropriately and rigorously?

Reviewer #2: No

4. Have the authors made all data underlying the findings in their manuscript fully available?

The [PLOS Data policy](http://track.editorialmanager.com/CL0/http:%2F%2Fwww.plosone.org%2Fstatic%2Fpolicies.action%23sharing/1/010f019e8bfe7a35-47bbabe2-dacb-43cb-ac1f-fe747377261d-000000/S4ijL3Pnn4e-CMlfvMJuG0SBt8CB9__BsyDULoZbPp8=258) requires authors to make all data underlying the findings described in their manuscript fully available without restriction, with rare exception (please refer to the Data Availability Statement in the manuscript PDF file). The data should be provided as part of the manuscript or its supporting information, or deposited to a public repository. For example, in addition to summary statistics, the data points behind means, medians and variance measures should be available. If there are restrictions on publicly sharing data—e.g. participant privacy or use of data from a third party—those must be specified.

Reviewer #2: Yes

5. Is the manuscript presented in an intelligible fashion and written in standard English?

PLOS ONE does not copyedit accepted manuscripts, so the language in submitted articles must be clear, correct, and unambiguous. Any typographical or grammatical errors should be corrected at revision, so please note any specific errors here.

Reviewer #2: Yes

6. Review Comments to the Author

Please use the space provided to explain your answers to the questions above. You may also include additional comments for the author, including concerns about dual publication, research ethics, or publication ethics. (Please upload your review as an attachment if it exceeds 20,000 characters)

**Reviewer #2: The authors have thoroughly addressed my previous comments, and most of my concerns have now been resolved. However, I do not think the interaction among sex, age and CCS (and coronary stenosis) has been sufficiently addressed. The relation between age and coronary atherosclerosis is highly influenced by sex, and vice versa. While the individual effects of age and sex on CCS have been analyzed in a multivariable analysis, evaluation of the combined or interactive impact of age and sex on CCS, rather than just their separate contributions, is required.
In response to my previous comment, the authors stated that the primary objective of the study is to investigate the impact of hypertension stages on CCS, not the relationship between age and sex. However, in the presence of the significant differences in age and sex among the hypertension stage groups, the study's conclusions may not be sufficiently robust without a thorough assessment of this issue
I recommend the authors to consider including an "age" × "sex" interaction term (and, if feasible, combinations of hypertension stage with age and sex) in the multivariable model, to reflect the different age–CCS trajectories between men and women. It would strengthen the robustness of the findings.**

**Author’s response:**

Thank you for these valuable comments. We agree that the relationship between age and coronary atherosclerosis may differ according to sex, and vice versa, and that evaluation of their potential interaction could strengthen the robustness of our findings. As suggested, we performed additional multivariable logistic regression analyses including an age-by-sex interaction term (age **×** sex) in the fully adjusted model.

For non-obstructive CAD, the age-by-sex interaction term was statistically significant (OR 0.965, 95% CI 0.947-0.984, p<0.001), indicating a significant interaction between age and sex in relation to non-obstructive CAD. However, after inclusion of the interaction term, stage 1 hypertension (OR 1.313, 95% CI 1.137-1.516, p<0.001) and stage 2 hypertension (OR 1.434, 95% CI 1.192-1.726, p<0.001) remained independently associated with non-obstructive CAD.

For obstructive CAD, the age-by-sex interaction term was not statistically significant (OR 1.000, 95% CI 0.963-1.038, p=0.998). Stage 2 hypertension remained significantly associated with obstructive CAD (OR 1.696, 95% CI 1.192-2.413, p=0.003).

These findings suggest that the primary association between hypertension stage and subclinical coronary atherosclerosis remained robust after accounting for the potential interaction between age and sex. As these additional analyses were conducted specifically to address the reviewer’s concern and did not materially change the main findings of the study, we have presented these results in this response for clarification.

Table R1. Multivariable logistic regression analyses including an age-by-sex interaction term

| Variables | Non-obstructive CAD | | | Obstructive CAD | |
| --- | --- | --- | --- | --- | --- |
|  | Multivariable analysis | | Multivariable analysis | | |
|  | Odds ratio (95% CI) | p-value | Odds ratio (95% CI) | | p-value |
| Age × Sex interaction | 0.965 (0.947 – 0.984) | <0.001 | 1.000 (0.963 – 1.038) | | 0.998 |
| Category of blood pressure |  | <0.001 |  | | 0.033 |
| Normal (reference) | 1 | – | 1 | | – |
| Elevated | 0.970 (0.795 – 1.183) | 0.761 | 1.305 (0.894 – 1.906) | | 0.168 |
| Stage 1 | 1.313 (1.137 – 1.516) | <0.001 | 1.261 (0.940 – 1.693) | | 0.122 |
| Stage 2 | 1.434 (1.192 – 1.726) | <0.001 | 1.696 (1.192 – 2.413) | | 0.003 |

* Covariates in the multivariable model include age, sex, obesity (body mass index ≥25 kg/m^2^), diabetes mellitus, hyperlipidemia, current smoking, family history of coronary artery disease, and the age-by-sex interaction term.

7. PLOS authors have the option to publish the peer review history of their article ([what does this mean?](https://track.editorialmanager.com/CL0/https:%2F%2Fjournals.plos.org%2Fplosone%2Fs%2Feditorial-and-peer-review-process%23loc-peer-review-history/1/010f019e8bfe7a35-47bbabe2-dacb-43cb-ac1f-fe747377261d-000000/maYg8Oy7zRFNXaUrF_X2-4bAwIo0qnI8uBXNOBgiiZY=258)). If published, this will include your full peer review and any attached files.


If you choose “no”, your identity will remain anonymous but your review may still be made public.


Do you want your identity to be public for this peer review? For information about this choice, including consent withdrawal, please see our [Privacy Policy](https://track.editorialmanager.com/CL0/https:%2F%2Fwww.plos.org%2Fprivacy-policy/1/010f019e8bfe7a35-47bbabe2-dacb-43cb-ac1f-fe747377261d-000000/UCSuLbc552hzrgToWSUU1yjjlWdNNX-D5AxuWtM0QCA=258).

Reviewer #2: No
