# Supplementary material for: Degree of hypertension and subclinical coronary atherosclerosis in asymptomatic individuals without cardiovascular disease
Source: PLoS One. 2026 Jul 10;21(7):e0353359. doi: 10.1371/journal.pone.0353359 (PMC13354002; doi:10.1371/journal.pone.0353359)
Supplement: Peer review history

# Peer review: Decision Letter 0

**DOI:** 10.1371/journal.pone.0353359.r001

**Authors:** Neftali Eduardo Antonio-Villa

25 Feb 2026

PONE-D-25-67701

Degree of hypertension and subclinical coronary atherosclerosis in asymptomatic individuals without cardiovascular disease

PLOS One

Dear Dr. Park,

Thank you for submitting your manuscript to PLOS ONE. After careful consideration, we feel that it has merit but does not fully meet PLOS ONE’s publication criteria as it currently stands. Therefore, we invite you to submit a revised version of the manuscript that addresses the points raised during the review process.

Please submit your revised manuscript by Apr 11 2026 11:59PM. If you will need more time than this to complete your revisions, please reply to this message or contact the journal office at plosone@plos.org. When you're ready to submit your revision, log on to https://www.editorialmanager.com/pone/ and select the 'Submissions Needing Revision' folder to locate your manuscript file.

Please include the following items when submitting your revised manuscript:

- A letter that responds to each point raised by the academic editor and reviewer(s). You should upload this letter as a separate file labeled 'Response to Reviewers'.
- A marked-up copy of your manuscript that highlights changes made to the original version. You should upload this as a separate file labeled 'Revised Manuscript with Track Changes'.
- An unmarked version of your revised paper without tracked changes. You should upload this as a separate file labeled 'Manuscript'.

If you would like to make changes to your financial disclosure, please include your updated statement in your cover letter. Guidelines for resubmitting your figure files are available below the reviewer comments at the end of this letter.

If applicable, we recommend that you deposit your laboratory protocols in protocols.io to enhance the reproducibility of your results. Protocols.io assigns your protocol its own identifier (DOI) so that it can be cited independently in the future. For instructions see: https://journals.plos.org/plosone/s/submission-guidelines#loc-laboratory-protocols. Additionally, PLOS ONE offers an option for publishing peer-reviewed Lab Protocol articles, which describe protocols hosted on protocols.io. Read more information on sharing protocols at https://plos.org/protocols?utm_medium=editorial-email&utm_source=authorletters&utm_campaign=protocols.

We look forward to receiving your revised manuscript.

Kind regards,

Neftali Eduardo Antonio-Villa, MD PhD

Academic Editor

PLOS One

Journal requirements:

When submitting your revision, we need you to address these additional requirements.

1. Please ensure that your manuscript meets PLOS ONE's style requirements, including those for file naming. The PLOS ONE style templates can be found at

https://journals.plos.org/plosone/s/file?id=wjVg/PLOSOne_formatting_sample_main_body.pdf and

https://journals.plos.org/plosone/s/file?id=ba62/PLOSOne_formatting_sample_title_authors_affiliations.pdf

2. Thank you for stating the following in the Funding Section of your manuscript:

“This research was supported by grants of the Ulsan University Hospital Research Grant (UUH-2024-11) and the Korea Health Technology R&D Project through the Korea Health Industry Development Institute (KHIDI), funded by the Ministry of Health & Welfare, Republic of Korea (HI23C0896). Additional support was provided by the Medical Data-Driven Hospital Support Project through the Korea Health Information Service (KHIS), funded by the Ministry of Health & Welfare, Republic of Korea. The funders had no role in the study design, data collection and analysis, decision to publish, or preparation of the manuscript.”

We note that you have provided funding information that is currently declared in your Funding Statement. However, funding information should not appear in the Acknowledgments section or other areas of your manuscript. We will only publish funding information present in the Funding Statement section of the online submission form.

Please remove any funding-related text from the manuscript and let us know how you would like to update your Funding Statement. Currently, your Funding Statement reads as follows:

“This research was supported by grants of the Ulsan University Hospital Research Grant (UUH-2024-11) and the Korea Health Technology R&D Project through the Korea Health Industry Development Institute (KHIDI), funded by the Ministry of Health & Welfare, Republic of Korea (HI23C0896). Additional support was provided by the Medical Data-Driven Hospital Support Project through the Korea Health Information Service (KHIS), funded by the Ministry of Health & Welfare, Republic of Korea. The funders had no role in the study design, data collection and analysis, decision to publish, or preparation of the manuscript.”

Please include your amended statements within your cover letter; we will change the online submission form on your behalf.

3. We note that you have indicated that there are restrictions to data sharing for this study. For studies involving human research participant data or other sensitive data, we encourage authors to share de-identified or anonymized data. However, when data cannot be publicly shared for ethical reasons, we allow authors to make their data sets available upon request. For information on unacceptable data access restrictions, please see http://journals.plos.org/plosone/s/data-availability#loc-unacceptable-data-access-restrictions.

Before we proceed with your manuscript, please address the following prompts:

a) If there are ethical or legal restrictions on sharing a de-identified data set, please explain them in detail (e.g., data contain potentially identifying or sensitive patient information, data are owned by a third-party organization, etc.) and who has imposed them (e.g., a Research Ethics Committee or Institutional Review Board, etc.). Please also provide contact information for a data access committee, ethics committee, or other institutional body to which data requests may be sent.

b) If there are no restrictions, please upload the minimal anonymized data set necessary to replicate your study findings to a stable, public repository and provide us with the relevant URLs, DOIs, or accession numbers. Please see http://www.bmj.com/content/340/bmj.c181.long for guidelines on how to de-identify and prepare clinical data for publication. For a list of recommended repositories, please see https://journals.plos.org/plosone/s/recommended-repositories. You also have the option of uploading the data as Supporting Information files, but we would recommend depositing data directly to a data repository if possible.

Please update your Data Availability statement in the submission form accordingly.

If the reviewer comments include a recommendation to cite specific previously published works, please review and evaluate these publications to determine whether they are relevant and should be cited. There is no requirement to cite these works unless the editor has indicated otherwise.

Additional Editor Comments:

After considering the revised manuscript and the comments from six external referees, the majority found the work relevant, well executed, and supported by interesting results. However, I have one major concern that the authors should address. A prior publication has examined a very similar question, differing primarily in the population studied (PMID: 37517130). The authors should therefore clearly delineate how the present study differs from that work and articulate the incremental value of extending the analysis to individuals with and without CVD, rather than focusing only on diabetes.

[Note: HTML markup is below. Please do not edit.]

Reviewers' comments:

Reviewer's Responses to Questions

**Comments to the Author**

1. Is the manuscript technically sound, and do the data support the conclusions?

The manuscript must describe a technically sound piece of scientific research with data that supports the conclusions. Experiments must have been conducted rigorously, with appropriate controls, replication, and sample sizes. The conclusions must be drawn appropriately based on the data presented.

Reviewer #1: Yes

Reviewer #2: Yes

Reviewer #3: Partly

Reviewer #4: Partly

Reviewer #5: Yes

Reviewer #6: Partly

**********

2. Has the statistical analysis been performed appropriately and rigorously?

Reviewer #1: No

Reviewer #2: Yes

Reviewer #3: Yes

Reviewer #4: Yes

Reviewer #5: Yes

Reviewer #6: Yes

**********

3. Have the authors made all data underlying the findings in their manuscript fully available?

The PLOS Data policy requires authors to make all data underlying the findings described in their manuscript fully available without restriction, with rare exception (please refer to the Data Availability Statement in the manuscript PDF file). The data should be provided as part of the manuscript or its supporting information, or deposited to a public repository. For example, in addition to summary statistics, the data points behind means, medians and variance measures should be available. If there are restrictions on publicly sharing data—e.g. participant privacy or use of data from a third party—those must be specified.

Reviewer #1: No

Reviewer #2: Yes

Reviewer #3: No

Reviewer #4: No

Reviewer #5: No

Reviewer #6: Yes

**********

4. Is the manuscript presented in an intelligible fashion and written in standard English?

PLOS ONE does not copyedit accepted manuscripts, so the language in submitted articles must be clear, correct, and unambiguous. Any typographical or grammatical errors should be corrected at revision, so please note any specific errors here.

Reviewer #1: Yes

Reviewer #2: Yes

Reviewer #3: Yes

Reviewer #4: Yes

Reviewer #5: Yes

Reviewer #6: Yes

**********

5. Review Comments to the Author

Please use the space provided to explain your answers to the questions above. You may also include additional comments for the author, including concerns about dual publication, research ethics, or publication ethics. (Please upload your review as an attachment if it exceeds 20,000 characters)

Reviewer #1: In this study Park and coworkers analyze the correlation between hypertension and extent of CAD in CCTA in a retrospective cohort. They find a correlation after correcting for other risk factors.

While the multivariate analysis is fully comprehensible, the other statistics remain vague.

All presented test test for a difference between at least 2 categories out of the 4. This makes the resuls somehow arbitrary, a p for trend would be more meaningfull, hypertension is ordinal and not categorical.

In remains unclear why two different test were used for categorical and ordinal data each.

There is date on calcium score that is not analyzed, paritcularly in this asymptomatic cohort calcium score is an important predictor. An analysis might give further insight.

Overall novelty and clinical usage of the data is limited.

Reviewer #2: The authors retrospectively investigated the degree of hypertension and non-obstructive and obstructive coronary disease in 7332 asymptomatic subjects without cardiovascular disease who underwent CCTA for a general health examination (mean age 52.8 ± 7.8 years, 63.8% male). Hypertension was graded as normal (<120/80 mmHg), elevated (120-129/ <80 mmHg), stage 1 hypertension (130-139/80-89 mmHg), and stage 2 hypertension (≥140/90 mmHg) according to ACC/AHA 2025 guidelines. Coronary artery calcium score (CACS) was classified as 0, 1-10, 11-100, 101-400, and >400, and stenosis ≥50% on CCTA was defined as obstructive CAD. The CACS grade and mean value of CACS were increased progressively with increasing hypertension stages. The degree of hypertension was also associated with the prevalence of obstructive CAD and non-obstructive CAD, and the stage 2 hypertension group showed a higher prevalence of both types of CAD compared with the other groups. Multivariate logistic regression analysis indicated that non-obstructive CAD was significantly associated with both stage 1 and stage 2 hypertension after adjustment for other risk factors, while obstructive CAD was associated with stage 2 hypertension, but not with stage 1 hypertension.

1) The major finding of the present study is that higher stages of hypertension are associated with higher CACS even in asymptomatic subjects. Similar findings were previously reported in several studies (e.g., Kang J, Atherosclerosis. 2019;282:188-195, Im TS, Int J Cardiovasc Imaging. 2014;30 Suppl 2:105-112, etc.). The authors should indicate the differences between this study and previous studies in the Discussion section. In particular, the authors should emphasize how their application of the new hypertension grading, according to the new ACC/AHA guidelines, distinguishes their findings from previous research.

2) What were the reasons for performing CCTA in 10,581 asymptomatic patients? Please indicate the major reasons for CCTA in this study cohort.

3) Although all continuous values are presented as mean ± standard deviation, those without normal distribution should be presented as median (interquartile range) for accurate representation.

4) Patients with a CACS of 0 have an extremely low risk of major adverse cardiovascular events (MACE) and all-cause mortality, with event rates consistently <1% per year. Those with a CAC score ≥1 have a significantly higher risk of cardiovascular events, with risk increasing as the score rises. Even minimal CACS (1-99) confers a borderline to intermediate 10-year risk (5-7.5%). Thus, it is clinically important to differentiate those with a CACS 0f 0 from those with CACS with ≥1. The authors should investigate the association between CACS=0 and hypertension grade.

5) There is a difference among the groups based on hypertension stages in several clinical factors (age, sex, BMI, etc.) and comorbidities. I recommend performing subgroup analyses according to these factors to explore whether they modify the association between hypertension grade and CACS or coronary stenosis.

6) Subjects of this study were divided into 5 groups as CACS 0, 1–10, 11–100, 101–400, >400. Although this stratification is acceptable, it may be helpful to additionally perform analyses using more commonly employed CACS categories (0, 1–99, 100–299, ≥300), which are frequently used in recent literature (see Slipczuk L, Blankstein R, Bucciarelli-Ducci C, et al. Circulation. 2025;152:e443-e466), to facilitate comparison with other studies.

Minor comments:

“Dyslipidemia” is a more suitable term than “hyperlipidemia”.

Reviewer #3: Manuscript: Degree of hypertension and subclinical coronary atherosclerosis in asymptomatic individuals without cardiovascular disease

General Assessment

This manuscript presents a large retrospective cross-sectional analysis of 7,332 asymptomatic individuals undergoing coronary computed tomography angiography (CCTA) as part of a health screening program. The authors investigate the association between blood pressure (BP) categories defined by contemporary ACC/AHA thresholds and the presence and severity of subclinical coronary artery disease (CAD).

The dataset is substantial, the imaging methodology appears appropriate, and the statistical analyses are coherent and generally robust. The findings demonstrate statistically significant graded associations between higher BP categories and both non-obstructive and obstructive CAD, consistent with established pathophysiologic expectations.

However, several important methodological and interpretative aspects require clarification to ensure that the manuscript’s conclusions are fully supported and appropriately framed. The overall direction of the findings is consistent with existing evidence, and the primary issues relate to framing and proportional interpretation rather than analytic validity.

1) Main Claims and Their Significance

The central claim of the manuscript is that an increase in BP category is associated with a greater prevalence and severity of subclinical CAD detected by CCTA in asymptomatic individuals without known cardiovascular disease.

This is a clinically and epidemiologically meaningful question. However, the manuscript currently presents the association as if it addresses an unresolved uncertainty in the field. In reality, the relationship between elevated BP and coronary atherosclerosis is well established epidemiologically. The study does not represent a paradigm shift, but rather provides large-scale imaging-based confirmation within a screening cohort using contemporary BP thresholds. In this context, the contribution of the present study is primarily incremental rather than transformative, which is entirely appropriate provided that it is positioned with precision.

To strengthen the manuscript, clarification is needed regarding the specific gap this study addresses. In particular, the manuscript would benefit from a more apparent distinction between confirmatory evidence and novel contribution, and from explicitly defining whether the added value lies in the screening population, the untreated BP phenotype, or the imaging-based assessment.

This refinement would enhance intellectual coherence without requiring additional data.

2) Definition of Exposure: Blood Pressure Phenotype vs Clinical Hypertension

A central conceptual issue concerns how “hypertension” is defined. BP categories are derived from measurements obtained at a single visit, using the average of the second and third readings. Additionally, individuals receiving antihypertensive therapy were excluded (as shown in the flow diagram). Therefore, the study does not evaluate clinically diagnosed hypertension as a chronic condition. Instead, it examines a blood pressure phenotype measured at a single encounter in untreated individuals. This distinction is critical, as clinical diagnosis of hypertension is inherently longitudinal and requires repeated measurements. Single-visit BP classification may lead to nondifferential misclassification. Excluding treated individuals removes a biologically and clinically important segment of the hypertensive population.

At the same time, this design creates a unique opportunity: it isolates the association between current hemodynamic exposure and coronary morphology, free from pharmacologic modification.

The manuscript would benefit from explicitly clarifying in the Methods that BP classification is based on single-visit measurement. Potential misclassification should be acknowledged in the Discussion, and language implying conclusions about diagnosed hypertension may need to be reconsidered. In addition, the implications of excluding treated individuals deserve further discussion, particularly with respect to biological interpretation and generalizability. This clarification would significantly improve conceptual precision.

3) Placement Within Existing Literature

The Discussion heavily references prospective and interventional trials (e.g., BP-lowering trials and prognostic imaging studies). While such references provide context, the present study is cross-sectional and observational. Data in the manuscript demonstrate association, not causation. They do not evaluate treatment response or long-term outcomes. Accordingly, a more precise separation between cross-sectional findings and interventional evidence is warranted. In particular, the manuscript should avoid implying that the present data directly support specific therapeutic strategies and instead frame conclusions at the level of vascular phenotype and subclinical remodelling rather than at the level of intervention. This will ensure proportionality between design and interpretation.

4) Effect Size and Clinical Interpretation

The adjusted odds ratios reported in the manuscript (approximately 1.3–1.7) indicate statistically significant associations between higher BP categories and the presence of non-obstructive and obstructive CAD. However, the clinical significance of these associations cannot be fully appreciated from odds ratios alone. To enhance interpretability, presenting absolute CAD prevalence across BP categories would be valuable. Reporting adjusted predicted probabilities derived from the multivariable model may further translate odds ratios into clinically interpretable estimates. Finally, statements implying substantial risk increase should be proportionate to the observed magnitude of association.

Providing absolute and adjusted risk estimates would substantially enhance clinical interpretability. This distinction is particularly relevant in large cohorts, where statistical significance may not fully reflect clinical magnitude.

5) Statistical Modelling Transparency

While the multivariable logistic regression models appear appropriate, the manuscript does not provide sufficient detail regarding model diagnostics and variable selection strategy. Greater transparency regarding statistical modelling would strengthen the manuscript. It would be helpful to indicate whether multicollinearity diagnostics (e.g., Variance Inflation Factor) were performed, to clarify the rationale for covariate selection, and to specify the statistical tests underlying the comparisons in Table 1. Given the ordinal structure of CAD severity, a brief justification for the choice of separate binary models would also be informative.

These clarifications would strengthen the methodological rigour of the manuscript without requiring substantial reanalysis.

6) Recruitment Period (2009–2020) and Temporal Heterogeneity

The recruitment period spans more than a decade. Between 2009 and 2020, substantial changes occurred in preventive cardiology and imaging: expansion of statin use, evolving lipid targets, increased BP awareness, and improvements in CCTA acquisition and reconstruction. Such temporal shifts may influence both the distribution of exposure and the detection and classification of CAD. It is unclear whether the year of examination was considered in the modelling and whether the associations were stable across time. Clarification of how the calendar year is handled in the analysis would strengthen confidence in the robustness of the findings. Consideration of temporal heterogeneity across the recruitment period, either analytically or within the Discussion, appears warranted.

This issue does not undermine the findings but warrants acknowledgement.

As with all observational cross-sectional analyses, residual confounding remains a plausible consideration, particularly given that cumulative BP exposure and certain lifestyle or socioeconomic factors may not be fully captured in the current dataset.

7) Transparency of Study Population

Although the flow diagram presents exclusion criteria, the textual Methods section does not comprehensively describe all inclusion and exclusion criteria. For reproducibility and clarity, the Study Population subsection should explicitly list all inclusion and exclusion criteria and provide a rationale for key exclusions, especially those related to antihypertensive therapy. Transparent reporting is essential for interpretability.

8) ASCVD Risk Score

The manuscript reports the calculation of 10-year ASCVD risk using pooled cohort equations. Additional clarification regarding the rationale for applying pooled cohort equations in this population would be appropriate. The role of ASCVD risk in the analysis, along with potential calibration limitations, merits a brief discussion.

9) Depth of CCTA Phenotyping

The analysis relies on stenosis-based classification and CACS categories. Contemporary CCTA platforms allow more detailed plaque characterisation. If plaque burden or composition data were unavailable due to retrospective constraints, this should be explicitly stated. Clarification regarding the availability of plaque burden or compositional metrics would be helpful. If such data were unavailable, this limitation should be explicitly acknowledged, particularly given that BP may correlate more closely with plaque burden than with binary stenosis thresholds.

10) Imaging-Based Vascular Phenotyping: Conceptual Refinement

This study has the potential to make a meaningful contribution to the concept of imaging-based vascular phenotyping. By focusing on untreated individuals and single-visit BP phenotype within a screening population, the study provides a snapshot of how current hemodynamic load relates to structural coronary remodelling. The Discussion would benefit from emphasising imaging-defined vascular phenotype, avoiding overextension into therapeutic inference, and framing the findings as characterisation of subclinical structural adaptation across BP strata. Such reframing would elevate the manuscript intellectually while remaining faithful to the data.

Final Recommendation

The study is methodologically sound and suitable for consideration in PLOS ONE. The requested revisions primarily concern clarification, proportional interpretation, and conceptual refinement. With appropriate revision, the manuscript would represent a methodologically sound and proportionately framed contribution within the scope of PLOS ONE.

Reviewer #4: Synopsys

This study evaluated the relationship between hypertension grade and subclinical coronary atherosclerosis in 7,332 asymptomatic individuals without known cardiovascular disease (mean age 52.8 ± 7.8 years; 63.8% men) who underwent CCTA. Participants were categorized as normal blood pressure (n=2,500), elevated (n=969), stage 1 hypertension (n=2,841), and stage 2 hypertension (n=1,022) according to ACC/AHA criteria. Coronary arteries were classified as normal, non-obstructive CAD (<50% stenosis), or obstructive CAD (≥50% stenosis). After adjustment for cardiovascular risk factors, stage 1 hypertension showed an association with non-obstructive CAD (aOR 1.335; 95% CI 1.156–1.541). Stage 2 hypertension showed associations with non-obstructive CAD (aOR 1.483; 95% CI 1.234–1.784) and obstructive CAD (aOR 1.696; 95% CI 1.194–2.409). Increasing hypertension category corresponded to higher prevalence of CAD.

General comment

1. The manuscript shows substantial overlap with the authors’ previous publication “Differential Impact of Degree of Hypertension on Subclinical Coronary Atherosclerosis in Asymptomatic Subjects With and Without Diabetes Mellitus” (DOI: 10.1016/j.amjcard.2023.07.036). The cohort appears to be essentially the same in size, demographics, and source population, and the core methodology (CCTA-based assessment of subclinical CAD and hypertension staging) is also largely unchanged. Although the present study does not stratify by diabetes status, the central message and direction of the associations are highly similar to those already reported. In its current form, the incremental value seems limited. To justify a separate publication, the authors should clearly state the degree of dataset overlap with prior work, explicitly delineate what is genuinely new in terms of hypothesis and analysis, and better articulate the additional clinical or scientific value provided by this study beyond their earlier report. Strengthening these aspects would help clarify the distinct contribution of the manuscript.

Reviewer #5: Park et al evaluated the correlation of arterial hypertension (HTN) and coronary atherosclerosis (CAD) assessed by computed tomography angiography (CTA) in a large cohort of asymptomatic patients. The main finding of the study is that stage 1 HTN was independently associated with non-obstructive CAD and stage 2 HTN with both non-obstructive and obstructive CAD.

This work was designed as a retrospective analysis of a large prospective cohort. The text is generally well written, easy to read and completely understandable. Methods, results and discussion sections are well described, presented and commented on. In any case, some questions need to be addressed.

-First of all, this study encompasses a large cohort of asymptomatic individuals undergoing CTA as part of “a general health examination”. As this was not a clinical trial and CTA is only indicated in selected high risk asymptomatic patients(1), a further explanation of its indication would be desirable.

-Regarding CTA analysis some additional data should be included. Calcium score was reported and analyzed by categories. However, it is known that the values are highly dependent on age and sex; therefore, an additional analysis considering the percentile(2) would be interesting. Current SCCT guidelines(3) recommend a quantitative stenosis grading of 5 categories and the use of the CAD-RADS 2.0 classification system(4). The limited classification of obstructive (<50%) and non-obstructive (>50%) CAD precludes a evaluation of the importance of HTN in the CAD burden. Thus, a detailed coronary stenosis grading should be performed and analyzed.

-Univariate as well as multivariate analysis reveal that not only HTN but almost all the rest of cardiovascular risk factors were associated with CAD in CTA. ASCVD risk score was ASCVD risk score was associated with the different categories of blood pressure, but it was included nor in the univariate neither in the multivariate analysis of CAD. Although the aim of the study was to determine the independent relevance of HTN, over the other risk factors, an specific analysis of ASCVD risk score should be performed.

In summary, this study reinforces the independent association of HTN with CAD evaluated by CTA. Nonetheless, aforementioned aspects related with patient population and methodology need a further explanation.

REFERENCES

1. Narula J, Chandrashekhar Y, Ahmadi A, Abbara S, Berman DS, Blankstein R, et al. SCCT 2021 Expert Consensus Document on Coronary Computed Tomographic Angiography: A Report of the Society of Cardiovascular Computed Tomography. J Cardiovasc Comput Tomogr. 2021;15(3):192-217.

2. McClelland RL, Chung H, Detrano R, Post W, Kronmal RA. Distribution of coronary artery calcium by race, gender, and age: results from the Multi-Ethnic Study of Atherosclerosis (MESA). Circulation. 2006;113(1):30-7.

3. Raff GL, Abidov A, Achenbach S, Berman DS, Boxt LM, Budoff MJ, et al. SCCT guidelines for the interpretation and reporting of coronary computed tomographic angiography. J Cardiovasc Comput Tomogr. 2009;3(2):122-36.

4. Cury RC, Leipsic J, Abbara S, Achenbach S, Berman D, Bittencourt M, et al. CAD-RADS 2.0 - 2022 Coronary Artery Disease-Reporting and Data System: An Expert Consensus Document of the Society of Cardiovascular Computed Tomography (SCCT), the American College of Cardiology (ACC), the American College of Radiology (ACR), and the North America Society of Cardiovascular Imaging (NASCI). J Cardiovasc Comput Tomogr. 2022;16(6):536-57.

Reviewer #6: This manuscript presents a large cross-sectional analysis of asymptomatic individuals undergoing CCTA and evaluates the association between hypertension severity and subclinical coronary atherosclerosis. The research question is clearly defined and clinically relevant. The sample size is substantial and imaging assessment appears rigorous. The findings are generally supported by the presented data.

However, several methodological aspects require clarification to ensure transparency and scientific rigor:

Antihypertensive medication status: The flow diagram suggests exclusion of individuals receiving antihypertensive therapy, yet the Methods section does not clearly describe how treated hypertension was handled. Clarification is needed regarding inclusion/exclusion criteria and classification of treated individuals, as this may influence interpretation.

Blood pressure classification: BP was measured during a single health screening visit. Although repeated measurements during the visit were averaged appropriately, discussion of potential misclassification (e.g., white coat effect or previously diagnosed hypertension) would strengthen interpretability.

Multivariable model specification: While the included covariates are clinically appropriate, additional transparency regarding model selection strategy, assessment of multicollinearity, and rationale for not including ASCVD risk score would improve methodological clarity.

Cross-sectional design: The study demonstrates association rather than causation. The Discussion would benefit from explicitly reinforcing this distinction.

Elevated BP category: The loss of statistical significance after adjustment warrants brief discussion to aid interpretation.

Overall, the manuscript addresses a relevant research question and is potentially suitable for publication after clarification of the above points.

**********

6. PLOS authors have the option to publish the peer review history of their article (what does this mean?). If published, this will include your full peer review and any attached files.

If you choose “no”, your identity will remain anonymous but your review may still be made public.

**Do you want your identity to be public for this peer review?**  For information about this choice, including consent withdrawal, please see our Privacy Policy.

Reviewer #1: No

Reviewer #2: No

Reviewer #3: **Yes:** Rafal Dankowski

Reviewer #4: No

Reviewer #5: No

Reviewer #6: No

**********

[NOTE: If reviewer comments were submitted as an attachment file, they will be attached to this email and accessible via the submission site. Please log into your account, locate the manuscript record, and check for the action link "View Attachments". If this link does not appear, there are no attachment files.]

To ensure your figures meet our technical requirements, please review our figure guidelines: https://journals.plos.org/plosone/s/figures

You may also use PLOS’s free figure tool, NAAS, to help you prepare publication quality figures: https://journals.plos.org/plosone/s/figures#loc-tools-for-figure-preparation.

NAAS will assess whether your figures meet our technical requirements by comparing each figure against our figure specifications.

# Author comment: Author response to Decision Letter 1

**DOI:** 10.1371/journal.pone.0353359.r002

1 Apr 2026

Thank you for the reviewer's and editor's valuable comments. We have carefully addressed all comments and provided detailed, point-by-point responses in the uploaded 'Response to Reviewers' file. All corresponding revisions have been incorporated into the revised manuscript.

Attachment Submitted filename: Response to Reviewers.docx

# Peer review: Decision Letter 1

**DOI:** 10.1371/journal.pone.0353359.r003

**Authors:** Neftali Eduardo Antonio-Villa, Neftali Eduardo Antonio-Villa

30 Apr 2026

PONE-D-25-67701R1

Degree of hypertension and subclinical coronary atherosclerosis in asymptomatic individuals without cardiovascular disease

PLOS One

Dear Dr. Park,

Thank you for submitting your manuscript to PLOS ONE. After careful consideration, we feel that it has merit but does not fully meet PLOS ONE’s publication criteria as it currently stands. Therefore, we invite you to submit a revised version of the manuscript that addresses the points raised during the review process.

Please submit your revised manuscript by Jun 14 2026 11:59PM. If you will need more time than this to complete your revisions, please reply to this message or contact the journal office at plosone@plos.org. When you're ready to submit your revision, log on to https://www.editorialmanager.com/pone/ and select the 'Submissions Needing Revision' folder to locate your manuscript file.

Please include the following items when submitting your revised manuscript:

- A letter that responds to each point raised by the academic editor and reviewer(s). You should upload this letter as a separate file labeled 'Response to Reviewers'.
- A marked-up copy of your manuscript that highlights changes made to the original version. You should upload this as a separate file labeled 'Revised Manuscript with Track Changes'.
- An unmarked version of your revised paper without tracked changes. You should upload this as a separate file labeled 'Manuscript'.

If you would like to make changes to your financial disclosure, please include your updated statement in your cover letter. Guidelines for resubmitting your figure files are available below the reviewer comments at the end of this letter.

If applicable, we recommend that you deposit your laboratory protocols in protocols.io to enhance the reproducibility of your results. Protocols.io assigns your protocol its own identifier (DOI) so that it can be cited independently in the future. For instructions see: https://journals.plos.org/plosone/s/submission-guidelines#loc-laboratory-protocols. Additionally, PLOS ONE offers an option for publishing peer-reviewed Lab Protocol articles, which describe protocols hosted on protocols.io. Read more information on sharing protocols at https://plos.org/protocols?utm_medium=editorial-email&utm_source=authorletters&utm_campaign=protocols.

As the corresponding author, your ORCID iD is verified in the submission system and will appear in the published article. PLOS supports the use of ORCID, and we encourage all coauthors to register for an ORCID iD and use it as well. Please encourage your coauthors to verify their ORCID iD within the submission system before final acceptance, as unverified ORCID iDs will not appear in the published article. *Only*  the individual author can complete the verification step; PLOS staff *cannot*  verify ORCID iDs on behalf of authors.

We look forward to receiving your revised manuscript.

Kind regards,

Neftali Eduardo Antonio-Villa, MD PhD

Academic Editor

PLOS One

Journal Requirements:

1. If the reviewer comments include a recommendation to cite specific previously published works, please review and evaluate these publications to determine whether they are relevant and should be cited. There is no requirement to cite these works unless the editor has indicated otherwise.

2. Please review your reference list to ensure that it is complete and correct. If you have cited papers that have been retracted, please include the rationale for doing so in the manuscript text, or remove these references and replace them with relevant current references. Any changes to the reference list should be mentioned in the rebuttal letter that accompanies your revised manuscript. If you need to cite a retracted article, indicate the article’s retracted status in the References list and also include a citation and full reference for the retraction notice.

**Additional Editor Comments:**

Please, address the comments raised by Reviewer 2

[Note: HTML markup is below. Please do not edit.]

Reviewers' comments:

Reviewer's Responses to Questions

**Comments to the Author**

1. If the authors have adequately addressed your comments raised in a previous round of review and you feel that this manuscript is now acceptable for publication, you may indicate that here to bypass the “Comments to the Author” section, enter your conflict of interest statement in the “Confidential to Editor” section, and submit your "Accept" recommendation.

Reviewer #2: (No Response)

Reviewer #3: All comments have been addressed

Reviewer #5: All comments have been addressed

**********

2. Is the manuscript technically sound, and do the data support the conclusions?

The manuscript must describe a technically sound piece of scientific research with data that supports the conclusions. Experiments must have been conducted rigorously, with appropriate controls, replication, and sample sizes. The conclusions must be drawn appropriately based on the data presented.

Reviewer #2: Partly

Reviewer #3: Yes

Reviewer #5: Yes

**********

3. Has the statistical analysis been performed appropriately and rigorously?

Reviewer #2: No

Reviewer #3: Yes

Reviewer #5: Yes

**********

4. Have the authors made all data underlying the findings in their manuscript fully available?

The PLOS Data policy requires authors to make all data underlying the findings described in their manuscript fully available without restriction, with rare exception (please refer to the Data Availability Statement in the manuscript PDF file). The data should be provided as part of the manuscript or its supporting information, or deposited to a public repository. For example, in addition to summary statistics, the data points behind means, medians and variance measures should be available. If there are restrictions on publicly sharing data—e.g. participant privacy or use of data from a third party—those must be specified.

Reviewer #2: Yes

Reviewer #3: No

Reviewer #5: Yes

**********

5. Is the manuscript presented in an intelligible fashion and written in standard English?

PLOS ONE does not copyedit accepted manuscripts, so the language in submitted articles must be clear, correct, and unambiguous. Any typographical or grammatical errors should be corrected at revision, so please note any specific errors here.

Reviewer #2: Yes

Reviewer #3: Yes

Reviewer #5: Yes

**********

6. Review Comments to the Author

Please use the space provided to explain your answers to the questions above. You may also include additional comments for the author, including concerns about dual publication, research ethics, or publication ethics. (Please upload your review as an attachment if it exceeds 20,000 characters)

Reviewer #2: I thank the authors for the revisions made in response to my previous comments. While the manuscript has improved in several aspects, there remain some important issues that need to be addressed.

1) In response to my comment regarding the previous studies by Kang and Im, the authors cited these studies and provided a detailed explanation of the differences between the present study and previous ones. However, these differences were described only as “Previous studies in asymptomatic populations have similarly reported higher coronary calcium burden with increasing blood pressure severity, although differences exist in BP classification systems and analytic approaches” in the text.I suggest that the authors incorporate a "shorter version" of their response (1) into the Discussion section, which will emphasize the new findings of this study and strengthen the conclusions.

2) In the response to my comment 2, the authors gave the detailed reasons and the background of performing coronary CCTA in 10,581 asymptomatic subjects. I am satisfied with this explanation, but the readers of this article may have the same question as I had. The authors should add a brief explanation of the background of performing CCTA as a part of general medical checkups, which will increase the readability of the article.

If CCTA is performed only in subjects who pay additional cost, it may introduce selection bias into the study population.

3) As previously pointed out, there are differences in many clinical characteristics, including age and sex, between the 4 groups (Table 1), and their influence must be analyzed carefully. The authors performed interaction analyses to evaluate whether the association between hypertension stage and CAD outcomes differed according to sex, age group, and obesity. They found a significant interaction between hypertension stage and age, but no significant interactions for sex or obesity in relation to non-obstructive CAD. However, these results may not be consistent with the well-known sex difference in CAC development, with women developing calcification approximately 10–20 years later than men at similar absolute CAC scores. Actually, this sex difference profoundly affects CAC interpretation, particularly in younger subjects where age-sex-race percentiles become essential for proper risk assessment.

The authors should provide a more detailed analysis of the influence of age and sex (or their interaction) on the development of CAC.

4) In the response to my comment on the CACS category, the authors provided the results of analysis based on the 4-group classification (CACS 0, 1-99, 100-299 and ≥300), which is a widely used classification in studies and in clinical practice, which was consistent with the results based on the 5-group classification. On the other hand, the authors provided the results of an analysis based on a 4-group classification (CACS 0, 1–99, 100–299, and ≥300), which is widely used in studies and clinical practice, and which was consistent with the results based on the 5-group classification. What is the rationale for dividing those with CACS 1–100 into 2 groups?

Reviewer #3: Thank you for the revised version of the manuscript. The authors have made a clear effort to address the methodological and conceptual points raised in the initial review, and the manuscript has improved as a result.

In particular, clarifying blood pressure classification based on single-visit measurements, along with an expanded discussion of its limitations, meaningfully strengthens the interpretability of the findings. The manuscript is now more transparent regarding the study design, statistical approach, and the scope of available imaging data, which improves overall clarity and reproducibility.

The revisions related to statistical modelling, study population, and imaging limitations are appropriate and sufficient for the scope of this analysis. The authors have also made a clear effort to align the interpretation of the results with the observational nature of the data, thereby improving the internal consistency of the manuscript.

One point that may still be worth keeping in mind is that some parts of the discussion are still somewhat more interpretative than the data allow. Given the cross-sectional design, maintaining consistently cautious language throughout the discussion and conclusions would further strengthen the manuscript and ensure that the interpretation remains fully aligned with the study design.

Overall, the manuscript now represents a methodologically sound and clearly presented analysis. I have no major concerns at this stage.

Reviewer #5: The authors have included the answers to the previously made questions along the article. A more detailed plaque analysis would be desirable, but unfortunately the authors argue that those data are no longer available. Moreover, there is still concern regarding the potential selection bias secondary to the autorreferal of the evaluated individuals to computed tomography angiography (CTA). In any case, even with those limitations the paper has been able to demonstrate that even at early stages, arterial hypertension is associated with coronary atherosclerosis. Therefore, I consider that this work is clinically relevant.

**********

7. PLOS authors have the option to publish the peer review history of their article (what does this mean?). If published, this will include your full peer review and any attached files.

If you choose “no”, your identity will remain anonymous but your review may still be made public.

**Do you want your identity to be public for this peer review?**  For information about this choice, including consent withdrawal, please see our Privacy Policy.

Reviewer #2: No

Reviewer #3: No

Reviewer #5: No

**********

[NOTE: If reviewer comments were submitted as an attachment file, they will be attached to this email and accessible via the submission site. Please log into your account, locate the manuscript record, and check for the action link "View Attachments". If this link does not appear, there are no attachment files.]

To ensure your figures meet our technical requirements, please review our figure guidelines: https://journals.plos.org/plosone/s/figures

You may also use PLOS’s free figure tool, NAAS, to help you prepare publication quality figures: https://journals.plos.org/plosone/s/figures#loc-tools-for-figure-preparation.

NAAS will assess whether your figures meet our technical requirements by comparing each figure against our figure specifications.

# Author comment: Author response to Decision Letter 2

**DOI:** 10.1371/journal.pone.0353359.r004

12 May 2026

Thank you for the reviewer's and editor's valuable comments. We have carefully addressed all comments and provided detailed, point-by-point responses in the

uploaded 'Response to Reviewers' file. All corresponding revisions have been incorporated into the revised manuscript.

Attachment Submitted filename: Response_to_Reviewers_auresp_2.docx

# Peer review: Decision Letter 2

**DOI:** 10.1371/journal.pone.0353359.r005

**Authors:** Neftali Eduardo Antonio-Villa, Neftali Eduardo Antonio-Villa, Neftali Eduardo Antonio-Villa

3 Jun 2026

PONE-D-25-67701R2

Degree of hypertension and subclinical coronary atherosclerosis in asymptomatic individuals without cardiovascular disease

PLOS One

Dear Dr. Park,

Thank you for submitting your manuscript to PLOS ONE. After careful consideration, we feel that it has merit but does not fully meet PLOS ONE’s publication criteria as it currently stands. Therefore, we invite you to submit a revised version of the manuscript that addresses the points raised during the review process.

Please submit your revised manuscript by Jul 18 2026 11:59PM. If you will need more time than this to complete your revisions, please reply to this message or contact the journal office at plosone@plos.org. When you're ready to submit your revision, log on to https://www.editorialmanager.com/pone/ and select the 'Submissions Needing Revision' folder to locate your manuscript file.

Please include the following items when submitting your revised manuscript:

- A letter that responds to each point raised by the academic editor and reviewer(s). You should upload this letter as a separate file labeled 'Response to Reviewers'.
- A marked-up copy of your manuscript that highlights changes made to the original version. You should upload this as a separate file labeled 'Revised Manuscript with Track Changes'.
- An unmarked version of your revised paper without tracked changes. You should upload this as a separate file labeled 'Manuscript'.

If you would like to make changes to your financial disclosure, please include your updated statement in your cover letter. Guidelines for resubmitting your figure files are available below the reviewer comments at the end of this letter.

If applicable, we recommend that you deposit your laboratory protocols in protocols.io to enhance the reproducibility of your results. Protocols.io assigns your protocol its own identifier (DOI) so that it can be cited independently in the future. For instructions see: https://journals.plos.org/plosone/s/submission-guidelines#loc-laboratory-protocols. Additionally, PLOS ONE offers an option for publishing peer-reviewed Lab Protocol articles, which describe protocols hosted on protocols.io. Read more information on sharing protocols at https://plos.org/protocols?utm_medium=editorial-email&utm_source=authorletters&utm_campaign=protocols.

As the corresponding author, your ORCID iD is verified in the submission system and will appear in the published article. PLOS supports the use of ORCID, and we encourage all coauthors to register for an ORCID iD and use it as well. Please encourage your coauthors to verify their ORCID iD within the submission system before final acceptance, as unverified ORCID iDs will not appear in the published article. *Only*  the individual author can complete the verification step; PLOS staff *cannot*  verify ORCID iDs on behalf of authors.

We look forward to receiving your revised manuscript.

Kind regards,

Neftali Eduardo Antonio-Villa, MD PhD

Academic Editor

PLOS One

Journal Requirements:

1. If the reviewer comments include a recommendation to cite specific previously published works, please review and evaluate these publications to determine whether they are relevant and should be cited. There is no requirement to cite these works unless the editor has indicated otherwise.

2. Please review your reference list to ensure that it is complete and correct. If you have cited papers that have been retracted, please include the rationale for doing so in the manuscript text, or remove these references and replace them with relevant current references. Any changes to the reference list should be mentioned in the rebuttal letter that accompanies your revised manuscript. If you need to cite a retracted article, indicate the article’s retracted status in the References list and also include a citation and full reference for the retraction notice.

[Note: HTML markup is below. Please do not edit.]

Reviewers' comments:

Reviewer's Responses to Questions

**Comments to the Author**

1. If the authors have adequately addressed your comments raised in a previous round of review and you feel that this manuscript is now acceptable for publication, you may indicate that here to bypass the “Comments to the Author” section, enter your conflict of interest statement in the “Confidential to Editor” section, and submit your "Accept" recommendation.

Reviewer #2: (No Response)

**********

2. Is the manuscript technically sound, and do the data support the conclusions?

The manuscript must describe a technically sound piece of scientific research with data that supports the conclusions. Experiments must have been conducted rigorously, with appropriate controls, replication, and sample sizes. The conclusions must be drawn appropriately based on the data presented.

Reviewer #2: Yes

**********

3. Has the statistical analysis been performed appropriately and rigorously?

Reviewer #2: No

**********

4. Have the authors made all data underlying the findings in their manuscript fully available?

The PLOS Data policy requires authors to make all data underlying the findings described in their manuscript fully available without restriction, with rare exception (please refer to the Data Availability Statement in the manuscript PDF file). The data should be provided as part of the manuscript or its supporting information, or deposited to a public repository. For example, in addition to summary statistics, the data points behind means, medians and variance measures should be available. If there are restrictions on publicly sharing data—e.g. participant privacy or use of data from a third party—those must be specified.

Reviewer #2: Yes

**********

5. Is the manuscript presented in an intelligible fashion and written in standard English?

PLOS ONE does not copyedit accepted manuscripts, so the language in submitted articles must be clear, correct, and unambiguous. Any typographical or grammatical errors should be corrected at revision, so please note any specific errors here.

Reviewer #2: Yes

**********

6. Review Comments to the Author

Please use the space provided to explain your answers to the questions above. You may also include additional comments for the author, including concerns about dual publication, research ethics, or publication ethics. (Please upload your review as an attachment if it exceeds 20,000 characters)

Reviewer #2: The authors have thoroughly addressed my previous comments, and most of my concerns have now been resolved. However, I do not think the interaction among sex, age and CCS (and coronary stenosis) has been sufficiently addressed. The relation between age and coronary atherosclerosis is highly influenced by sex, and vice versa. While the individual effects of age and sex on CCS have been analyzed in a multivariable analysis, evaluation of the combined or interactive impact of age and sex on CCS, rather than just their separate contributions, is required.

In response to my previous comment, the authors stated that the primary objective of the study is to investigate the impact of hypertension stages on CCS, not the relationship between age and sex. However, in the presence of the significant differences in age and sex among the hypertension stage groups, the study's conclusions may not be sufficiently robust without a thorough assessment of this issue

I recommend the authors to consider including an "age" × "sex" interaction term (and, if feasible, combinations of hypertension stage with age and sex) in the multivariable model, to reflect the different age–CCS trajectories between men and women. It would strengthen the robustness of the findings.

**********

7. PLOS authors have the option to publish the peer review history of their article (what does this mean?). If published, this will include your full peer review and any attached files.

If you choose “no”, your identity will remain anonymous but your review may still be made public.

**Do you want your identity to be public for this peer review?**  For information about this choice, including consent withdrawal, please see our Privacy Policy.

Reviewer #2: No

**********

[NOTE: If reviewer comments were submitted as an attachment file, they will be attached to this email and accessible via the submission site. Please log into your account, locate the manuscript record, and check for the action link "View Attachments". If this link does not appear, there are no attachment files.]

To ensure your figures meet our technical requirements, please review our figure guidelines: https://journals.plos.org/plosone/s/figures

You may also use PLOS’s free figure tool, NAAS, to help you prepare publication quality figures: https://journals.plos.org/plosone/s/figures#loc-tools-for-figure-preparation.

NAAS will assess whether your figures meet our technical requirements by comparing each figure against our figure specifications.

# Author comment: Author response to Decision Letter 3

**DOI:** 10.1371/journal.pone.0353359.r006

9 Jun 2026

We have carefully addressed the remaining reviewer comment by performing additional analyses including an age-by-sex interaction term. Detailed responses and supporting results are provided in the uploaded "Response to Reviewers" file.

Attachment Submitted filename: Response_to_Reviewers_auresp_3.docx

# Editor report: Decision Letter 3

**DOI:** 10.1371/journal.pone.0353359.r007

**Authors:** Neftali Eduardo Antonio-Villa, Neftali Eduardo Antonio-Villa, Neftali Eduardo Antonio-Villa, Neftali Eduardo Antonio-Villa

23 Jun 2026

Degree of hypertension and subclinical coronary atherosclerosis in asymptomatic individuals without cardiovascular disease

PONE-D-25-67701R3

Dear Dr. Park,

We’re pleased to inform you that your manuscript has been judged scientifically suitable for publication and will be formally accepted for publication once it meets all outstanding technical requirements.

Within one week, you’ll receive an e-mail detailing the required amendments. When these have been addressed, you’ll receive a formal acceptance letter and your manuscript will be scheduled for publication.

An invoice will be generated when your article is formally accepted. Please note, if your institution has a publishing partnership with PLOS and your article meets the relevant criteria, all or part of your publication costs will be covered. Please make sure your user information is up-to-date by logging into Editorial Manager at Editorial Manager® and clicking the ‘Update My Information' link at the top of the page. For questions related to billing, please contact billing support.

If your institution or institutions have a press office, please notify them about your upcoming paper to help maximize its impact. If they’ll be preparing press materials, please inform our press team as soon as possible -- no later than 48 hours after receiving the formal acceptance. Your manuscript will remain under strict press embargo until 2 pm Eastern Time on the date of publication. For more information, please contact onepress@plos.org.

Kind regards,

Neftali Eduardo Antonio-Villa, MD PhD

Academic Editor

PLOS One

Additional Editor Comments (optional):

I want to congratulate the authors on the extensive work in revising the manuscript. The conclusions and results have substantially improved since the original submission. I can now proceed to recommend this work for publication.

Reviewers' comments:

# Editor report: Acceptance letter

**DOI:** 10.1371/journal.pone.0353359.r008

**Authors:** Neftali Eduardo Antonio-Villa, Neftali Eduardo Antonio-Villa, Neftali Eduardo Antonio-Villa, Neftali Eduardo Antonio-Villa

PONE-D-25-67701R3

PLOS One

Dear Dr. Park,

I'm pleased to inform you that your manuscript has been deemed suitable for publication in PLOS One. Congratulations! Your manuscript is now being handed over to our production team.

At this stage, our production department will prepare your paper for publication. This includes ensuring the following:

* All references, tables, and figures are properly cited

* All relevant supporting information is included in the manuscript submission,

* There are no issues that prevent the paper from being properly typeset

You will receive further instructions from the production team, including instructions on how to review your proof when it is ready. Please keep in mind that we are working through a large volume of accepted articles, so please give us a few days to review your paper and let you know the next and final steps.

Lastly, if your institution or institutions have a press office, please let them know about your upcoming paper now to help maximize its impact. If they'll be preparing press materials, please inform our press team within the next 48 hours. Your manuscript will remain under strict press embargo until 2 pm Eastern Time on the date of publication. For more information, please contact onepress@plos.org.

You will receive an invoice from PLOS for your publication fee after your manuscript has reached the completed accept phase. If you receive an email requesting payment before acceptance or for any other service, this may be a phishing scheme. Learn how to identify phishing emails and protect your accounts at https://explore.plos.org/phishing.

If we can help with anything else, please email us at customercare@plos.org.

Thank you for submitting your work to PLOS ONE and supporting open access.

Kind regards,

PLOS ONE Editorial Office Staff

on behalf of

Dr. Neftali Eduardo Antonio-Villa

Academic Editor

PLOS One
